# PSMB10 maintains the stemness of chemotherapeutic drug-resistant leukemia cells by inhibiting senescence and cytotoxic T lymphocyte-mediated killing in a ubiquitinated degradation manner

**DOI:** 10.1186/s13046-025-03420-9

**Published:** 2025-06-03

**Authors:** Xiao Ma, Yingnan Li, Di Wang, Jialan Niu, Qian Li, Ying Chen, Mengyuan Wang, Jin Wen, Chenxi Liao, Nan Wang, Xiaolan Zhang, Jiwei Chang, Jiayi Yang, Lei Li, Jing Zou, Danyue Peng, Lingbo Liu

**Affiliations:** 1 Institute of Hematology, Union Hospital, Tongji Medical College, Huazhong University of Science and Technology, Wuhan, 430022 China; 2 Department of Pediatrics, Union Hospital, Tongji Medical College, Huazhong University of Science and Technology, Wuhan, 430022 China; 3 State Key Laboratory of Oral & Maxillofacial Reconstruction and Regeneration, Wuhan University, Wuhan, 430079 China; 4 College of Life Sciences, Wuhan University, Wuhan, 430072 China

**Keywords:** Acute myeloid leukemia, Leukemia stem cells, Chemotherapeutic drug resistance, Immunoproteasome, PSMB10, Senescence regulation, Immune escape

## Abstract

**Background:**

Drug resistance and relapse are still major challenges in acute myeloid leukemia (AML) because of the inability to effectively eradicate leukemia stem cells (LSCs). Senescence induction combined with immune killing may offer promising strategies for LSC eradication. However, whether and how drug-resistant LSCs retain stemness via senescence and immune regulation remains unknown.

**Methods:**

The immunoproteasome subunit *PSMB10* expression levels were analyzed by single-cell RNA-seq data, along with the bioinformatics analysis of publicly available AML datasets, and quantified using RT-qPCR and flow cytometry (FCM) analysis on clinical samples from AML patients. The cellular senescence was evaluated by the assays of cell proliferation, cell cycle, senescence-associated β-galactosidase activity, and senescence-associated secretory phenotype factors. In vitro T-cell killing assay was played to determine immune escape reprogramming of AML cells. FCM was conducted to estimate intracellular drug concentration and cellular apoptosis rates. Human AML xenografts and *PSMB10* knockout syngeneic mouse bone marrow transplantation models were utilized to investigate the function of PSMB10. Various techniques were employed for mechanism studies, including Lentivirus transduction or siRNA transfection, western blotting, co-immunoprecipitation assays, luciferase reporter assays, polysome profiling assays, quantitative proteomics, etc.

**Results:**

*PSMB10* mRNA was significantly upregulated in the surviving nonsenescent LSCs, exhibiting a 13-fold increase compared to senescent LSCs following chemotherapy. The specific high expression of PSMB10 in post-chemotherapy nonsenescent LSCs predicts a poor AML prognosis. The genetic inactivation of *PSMB10* resulted in increased senescence and cytotoxic T lymphocyte (CTL) killing, as well as increased intracellular drug concentrations and drug-induced cellular senescence in different types of human AML cells, which also impeded human and murine leukemia initiation and stemness maintenance in vivo with a 19-fold decrease in the frequency of human LSCs and a 7.6-fold decrease of drug-resistant mouse LSCs, while normal hematopoietic cells remained unaffected. Mechanistically, the downregulation of PSMB10 boosted SLC22A16-mediated drug endocytosis and further induced chemotherapy drug-mediated senescence through the RPL6/RPS6-MDM2-P21 pathway in AML cells. Additionally, downregulating PSMB10 also impeded MHC-I protein degradation-induced escape of CTL killing.

**Conclusions:**

PSMB10 is a key candidate molecular target for eradicating drug-resistant LSCs via senescence and immune regulation.

**Supplementary Information:**

The online version contains supplementary material available at 10.1186/s13046-025-03420-9.

## Introduction

Despite available treatments, over 70% of patients with AML cannot survive over 5 years owing to drug resistance and relapse, which are rooted in the inability to eradicate LSCs [1–3]. LSCs are a low-frequency subpopulation of leukemia cells that initiate and maintain AML [4, 5]. Acute promyelocytic leukemia (APL) is the only AML subtype that can be effectively cured with retinoic acid and/or arsenic trioxide, in which functional promyelocytic leukemia-p53 axis-induced senescence is required to eradicate LSCs [6–8]. We also found that decreased miR-34c-5p and increased RAB27B levels in non-APL-derived LSCs induce resistance to senescence and stemness maintenance [9, 10]. These findings suggest that restarting cell senescence could be an effective way to promote the clearance of LSCs. However, it has been observed that some senescent tumor cells could regain stemness, potentially leading to disease relapse [11]. Therefore, we speculate that the immune system should play a critical role in further eliminating senescent AML cells to achieve a cure for leukemia.

By analyzing single-cell RNA-seq data from bone marrow (BM) cells of AML patients [12], we found that PSMB10, a subunit of the immunoproteasome, was especially highly expressed in surviving postchemotherapy nonsenescent LSCs. A well-known function of immunoproteasome is to produce peptides for recognition by CD8^+^ T cells [13], and the knowledge of PSMB10 in AML is limited to the fact that its overexpression is linked to adverse overall survival in patients with AML [14]. However, whether and how increased *PSMB10* expression in surviving drug-resistant nonsenescent LSCs is involved in their stemness maintenance remains poorly understood.

Here, we found that increased PSMB10 expression in LSCs is associated with a poor prognosis in AML patients. The increased PSMB10 promotes the stemness maintenance of chemotherapeutic drug-resistant leukemia cells via the inhibition of SLC22A16-mediated drug endocytosis and RPL6/RPS6-MDM2-P21 pathway-initiated senescence, as well as the MHC-I protein degradation-induced escape of CTL killing.

## Methods

### Cell lines

The human acute leukemia cell line KG-1a was cultured in RPMI-1640 medium (Gibco, USA) supplemented with 20% FBS (Gibco) and 1% penicillin/streptomycin (Gibco). THP-1 cells were cultured in RPMI-1640 medium supplemented with 10% FBS and 1% penicillin/streptomycin. KG-1a and THP-1 cell lines were obtained from the American Type Culture Collection (ATCC) Institute. HEK293T cells were cultured in DMEM (Gibco) supplemented with 10% FBS and 1% penicillin/streptomycin (Gibco) and were obtained from the ATCC Institute. All the cell lines were authenticated via short tandem repeat profiling and tested for mycoplasma contamination yearly using a Mycoplasma Stain Assay Kit (Beyotime, China).

### AML patient and healthy individual samples

BM aspirates from AML patients and healthy individuals were obtained from Wuhan Union Hospital. Informed consent was obtained from all the patients in advance, and all the samples were approved by the Ethics Committee of Huazhong University of Science and Technology. BM mononuclear cells were acquired via density gradient centrifugation, and CD34^+^ stem cells were further separated via a CD34 microbead kit (Miltenyi, Germany). AML patient-derived CD34^+^ cells were cultured in StemSpan SFEM (09605, StemCell Technologies) supplemented with recombinant human IL3 (20 ng/mL, 200–03), FLT3-ligand (100 ng/mL, 300–19), TPO (50 ng/mL, 300–18), SCF (100 ng/mL, 300–07) (PeproTech, USA), and 1% penicillin/streptomycin [9, 15].

### Mice

C57BL/6 J (CD45.2) background *Psmb10*^*−/−*^ mice were purchased from Bestcell (Wuhan, China), and 8–10-week-old C57BL/6 J (CD45.2, Beijing Vital River Laboratory) mice were used for MLL-AF9 leukemia mouse transplantation model. NOD/ShiLtJGpt-*Prkdc*^em26Cd52^*Il2rg*^em26Cd22^/Gpt (NCG, strain no. T001475) mice were purchased from GemPharmatech (Nanjing, China), and NCG mice aged 6–8 weeks were used for AML xenograft model transplantation. All the mice were fed humanely, and approval was acquired from the Animal Care and Ethics Committee of Huazhong University of Science and Technology.

### Lentivirus transduction

shRNA constructs against PSMB10, RPL6, RPS6, and MHC-I were generated by Genomeditech Company (Shanghai, China) and Obio Company (Shanghai, China). All the target sequences are listed in Supplementary Table 1. For the PSMB10 rescue experiment and PSMB10 overexpression assay, the PSMB10 cDNA fragment was inserted into a lentivirus generated by the Obio Company (Shanghai, China). KG-1a and THP-1 cells were cultured overnight before transfection. Then, the cells were transfected with the corresponding lentiviruses [multiplicity of infection (MOI) = 80] in a medium containing 8 μg/ml polybrene and centrifuged at 2200 rpm for 1.5 h. At 18 h after transfection, the cells were resuspended in fresh medium, and positive control cells were selected with puromycin (4 μg/ml for KG-1a and 2 µg/ml for THP-1) or blasticidin (10 µg/ml for both KG-1a and THP-1) at 48 h after lentivirus transfection.

### siRNA transfection

PSMB10 siRNA and negative control were purchased from the Obio Company (Shanghai, China). These oligos worked at a final concentration of 100 nmol/l. A total of 2 × 10^6^ primary AML BM CD34^+^ cells (FAB, M0/M1/M2) or mononuclear cells (FAB, M5) were transfected via electroporation via an Amaxa® Cell Line Nucleofector® Kit L (Lonza, Swiss) by Amaxa Nucleofector II (Lonza) under program V-001 [10].

### Intracellular molecule staining

For intracellular staining of PSMB10, we first labeled BM mononuclear cells from AML patients with an anti-human FITC‒conjugated anti-CD34 antibody for 30 min at 4 °C. The cells were subsequently washed with precooled phosphate-buffered saline (PBS), resuspended in 100 µl of BD Cytofix/Cytoperm solution (554,722, BD) per 1 × 10^6^ cells, and incubated for 20 min at 4 °C. After being washed twice with Perm/Wash buffer (554,723, BD), the cells were thoroughly resuspended in 100 µl of Perm/Wash buffer containing PSMB10 primary antibody (ab183506, Abcam) at a dilution ratio of 1:250. Then, the cells were incubated at 4 °C for 30 min in the dark. After being washed twice with BD Perm/Wash buffer, the cells were resuspended in 100 µl of Perm/Wash buffer containing APC-labeled secondary antibody (M213811, Abmart) and incubated at 4 °C for 30 min in the dark. Finally, these cells were washed three times with precooled PBS and analyzed via FCM.

### Drug preparation and treatment

Cytarabine (147–94-4, MCE), daunorubicin (23,541–50-6, MCE), and adriamycin (25,316–40-9, MCE) were prepared in PBS, protected from light, and stored at -80 °C. For senescence induction, AML cell lines were exposed to cytarabine (AraC) at a final concentration of 0.2 µg/ml in growth medium for a duration of 3 days.

### Intracellular daunorubicin concentration assay

To study the drug resistance of AML cells, we exploited the fluorescence property of daunorubicin (DNR), which can be detected by FCM through the PE channel [16]. We incubated 1 × 10^6^ shCTRL- and shPSMB10-transduced THP-1 cells with 0.25 µg/ml daunorubicin (23,541–50-6, MCE) for 1 h at 37 °C [17]. Then, the cells were washed with PBS to remove daunorubicin from the medium, which was replaced with a fresh medium, for another 7 h. The first hour of coculture was considered as the process of daunorubicin uptake, followed by a daunorubicin efflux process after the first hour [18]; therefore, we continuously monitored the mean fluorescence intensity (MFI) of intracellular daunorubicin per hour via FCM via a BD LSRFortessaTM X-20 special order product (Becton), and the data were analyzed with FlowJo software.

### Cell proliferation assay

For the CCK-8 assay, 1 × 10^4^ THP-1 or KG-1a cells were cultured in a 96-well plate with 100 μl of complete medium. Cell viability was measured at days 0, 1, 2, 3, and 4 via a CCK-8 kit (C6005, NCM Biotech). For the EdU assay, EdU (C0081S, Beyotime) was added to the culture medium at a final concentration of 10 µM for 2 h, after which the cells were harvested and washed with PBS twice. Next, the cells were fixed, permeabilized, and dyed with a Click reaction mixture. Finally, the cells were resuspended in PBS and analyzed by FCM using a BD LSRFortessaTM X-20 special order product (Becton), and the data were analyzed with FlowJo software.

### Cell cycle assay

A Cell Cycle Analysis Kit (C1052, Beyotime) was used to assess the cell cycle. Briefly, the cells were collected and fixed in 70% ethanol at 4 °C overnight. The next day, the fixed pellets were washed with PBS and then incubated in a staining working solution containing RNase A and PI at 37 °C for 30 min. After incubation, the samples were assayed via FCM using a BD LSRFortessaTM X-20 special order product (Becton), and the data were analyzed with FlowJo software.

### SA-β-Gal assay

The SA-β-Gal Staining Kit (C0602, Beyotime) was used to detect SA-β-Gal activity in the cells. Similarly, the cells were collected and washed once with PBS, fixed with 1 ml of a β-galactosidase staining fixator, and fixed at room temperature for 15‒20 min. After fixation, the cells were washed three times with PBS and stained with a working solution at 37 °C for 48 h. Then, images were captured with an inverted fluorescence microscope (Olympus). For FCM analysis, SA-β-Gal activity was tested via a cellular senescence detection kit (SPiDER-βGal, Dojindo, Japan). The cultured cells were collected and incubated with bafilomycin A1 at 37 °C for 30 min. Next, the cells were incubated with SPiDER-β-Gal working solution at 37 °C for another hour according to the manufacturer’s instructions. The MFI was subsequently assayed via FCM via a BD LSRFortessaTM X-20 special order product (Becton), and the data were analyzed with FlowJo software.

### Apoptosis assay

A PE-Annexin V/7-AAD Apoptosis Detection Kit (559,763, BD) was used following the manufacturer’s instructions. Briefly, cultured cells were gently collected and washed twice with cold PBS, resuspended in 100 µl of binding buffer, stained with PE-Annexin V and 7AAD on ice for 15 min, and immediately subjected to flow cytometric analysis via a BD LSRFortessaTM X-20 special order product (Becton).

### RNA extraction, cDNA synthesis and RT-qPCR

AML cells were lysed with TRIzol reagent (TaKaRa, Japan), and total RNA was isolated via chloroform and isopropyl alcohol following standard protocols. For cDNA synthesis, 1 µg of total RNA was used for reverse transcription in a 20 μl reaction mixture with PrimeScript RT Master Mix (TaKaRa) at 37 °C for 15 min, 85 °C for 5 s, and 4 °C hold. Real-time fluorescence quantitative PCR (RT-qPCR) was performed with TB Green Premix Ex TaqII (TaKaRa) master mix in a StepOne Fast RT-qPCR system (Applied Biosystems), and the program was 40 cycles of running at 95 °C for 30 s, 95 °C for 10 s, and 60 °C for 30 s. GAPDH was used as an endogenous control, and the relative gene expression was calculated via the 2-ΔΔCt method normalized to GAPDH expression. All the primers used are listed in Supplementary Table 2.

### Co-IP analysis

THP-1 cells were collected and lysed in immunoprecipitation lysis buffer (25 mM Tris–HCl, 150 mM NaCl, 1 mM EDTA, and 1% NP-40) (G2038, Servicebio). The supernatants of the protein lysates were incubated with the following indicated antibodies at 4 °C for 1 h: anti-PSMB10 antibody (sc-133236, Santa Cruz, mouse), anti-RPL6 antibody (A303-587A-T, Thermo Fisher Scientific, rabbit), anti-RPS6 antibody (sc-74459, Santa Cruz, mouse), anti-MDM2 antibody (sc-965, Santa Cruz, mouse), anti-P21 antibody (2947, Cell Signaling Technology, rabbit), anti-MHC-I antibody (sc-32235, Santa Cruz, mouse), and anti-SLC22A16 antibody (sc-390056, Santa Cruz, mouse). Then, Protein A/G Plus-agarose (sc-2003, Santa Cruz) was added to the samples, which were subsequently incubated at 4 °C overnight. The beads were washed at least three times with lysis buffer. The bound proteins were separated via SDS‒polyacrylamide gel electrophoresis and identified via WB analysis with the corresponding antibodies.

### Protein extraction and immunoblot analysis

For total protein extraction, the washed cell pellets were lysed in RIPA buffer (HY-K1001, MCE) supplemented with 5 mM EDTA, 1 × Halt phosphatase inhibitor cocktail (78,420, Thermo Fisher Scientific), and 1 × Halt protease inhibitor cocktail (78,429, Thermo Fisher Scientific) on ice for 20 min, sonicated, and centrifuged at 20,000 × g at 4 °C for 15 min. The protein concentration was quantified with a BCA kit (G2026, Servicebio). Protein lysates were diluted with Protein Sample Loading Buffer (denaturing, non-reducing, 5 ×) (LT103, Epizyme) and denatured at 95 °C for 10 min. Western blotting was conducted as previously described. Briefly, equal amounts of protein extracts (20–50 mg) were separated by 6%-10% SDS‒PAGE gels and onto polyvinylidene difluoride (PVDF) membranes. The blots were blocked with 5% nonfat milk in phosphate buffered saline with 0.5% Tween-20 (PBST) and incubated with diluted primary antibodies in 1% (w/v) BSA in PBST overnight at 4 °C. Then, membranes were washed with PBST, incubated with a secondary antibody, and detected by Pierce ECL Western Blotting Substrate (PI32106, Thermo Fisher Scientific) or GE Healthcare Amersham ECL Prime Western Blotting Detection Reagent (45,010,090, GE Healthcare). The following primary antibodies were used for Western blotting: anti-GAPDH (1:5000, 10,494–1-AP, Proteintech), anti-Lamin B1 antibody (1:1000, A1910, ABclonal), anti-Cyclin D1 antibody (1:1000, A19038, ABclonal), Anti-CDK4 antibody (1:1000, ab137675, Abcam), anti-PSMB10 (1:1000, ab183506, Abcam), anti-RPL6 (1:2000, 15,387–1-AP, Proteintech), anti-RPS6 (1:5000, ab215214, Abcam), anti-MDM2 (1:1000, ab259265, Abcam), anti-P21 (1:1000, 2947, Cell Signaling Technology), anti-Ub (1:1000, PTM-7311, PTM Bio), anti-MHC-I (1:1000, ab225636, Abcam), anti-SLC22A16 (1:400, PA5-69,281, Invitrogen), anti-Cyclin E2 (1:1000, F2373, Selleck), anti-CDK2(1:1000, F0022, Selleck), anti-CDK6 (1:1000, F0372, Selleck), anti-Phospho-Rb (1:1000, F0302, Selleck) and anti-β-tubulin (1:8000, AC021, ABclonal). Secondary antibodies used in this study include Horseradish Peroxidase (HRP)-Conjugated Goat Anti-Mouse IgG H&L (HRP) (ab6789, Abcam) and Goat Anti-Rabbit IgG H&L (HRP) (ab6721, Abcam).

### In vitro T-cell killing assay

For the coculture assay, we isolated peripheral blood mononuclear cells (PBMNCs) from healthy donors via Ficoll separation (Ficoll Paque Plus, 1,714,444,003, Cytiva). Then, CD3 T cells were enriched from the above PBMNCs with a human CD3 selection kit (17,851, StemCell Technologies), maintained in 10% FBS RPMI-1640, activated with purified anti-human CD3 (317,326, Biolegend), and purified with anti-human CD28 (302,934, Biolegend) and recombinant human IL-2 for 5‒7 days. The CD3 antibody was precoated in 96-well cell culture plates overnight at 4 °C at a concentration of 10 µg/ml in PBS, while CD28 and rhIL-2 were added directly to the culture medium at final concentrations of 5 µg/ml and 50 U/ml, respectively. Moreover, both shCTRL and shPSMB10 KG-1a and THP-1 cells (1 × 10^5^ cells/well) were mixed with activated CD3 T cells at the indicated ratios and cocultured in 12-well plates for 18 h [19]. Afterward, the cells were collected, and the degree of apoptosis in the GFP^+^ AML cells was analyzed via FCM.

### Plasmid transformation and extraction experiments

For plasmid transformation, 100 µl of the competent cells were thawed on ice, and then, 50 µg of MLL-AF9-GFP plasmid was added and gently mixed. The system was immediately thermally activated in a water bath at 42 °C for 45 s after being kept on ice for 30 min and then kept on ice for another 30 min. Subsequently, 900 µl of Luria–Bertani (LB) medium was added, and the mixture was shaken at 200 rpm/min for 1 h on a 37 °C shaker. The bacterial mixture was then evenly spread onto an LB plate containing ampicillin resistance and incubated at 37 °C for 12–16 h. Monoclonal colonies were selected and cultured in an ampicillin-resistant LB medium and expanded by shaking at 200 rpm/min for 16 h on a 37 °C shaker. Finally, a high concentration of plasmid was extracted from the bacterial mixture via a plasmid extraction kit (Y1919, Tiangen).

### Retrovirus production

HEK239T cells were seeded in a 10-cm cell culture dish, and when the cells covered 60%-80% of the bottom of the plate, retroviral packaging was performed. Lipofectamine 2000 reagent (TL201-01, Vazyme) was incubated with Opti-MEM I reduced serum medium (Gibco), and HEK239T cells were transfected with 9 µg of the MSCV-MLL-AF9-IRES-GFP-encoding plasmid and 3 µg of the pCL-ECO packaging plasmid. Twelve hours after transfection, the medium was replaced with 10 ml of fresh medium. The supernatant containing the viruses was collected at 48 and 72 h, filtered through a 0.45 µm cellulose acetate filter, and used immediately or preserved at -80 °C for short-term storage.

### Proteomic analysis

For proteomic analysis, 5 × 10^7^ shCTRL and shPSMB10 cells were collected and quantified via iTRAQ by Novogene Company (China). Proteins with FC ≥ 1.2 and a q value < 0.05 were considered significant. InterproScan software was used for GO and IPR functional annotation (including the Pfam, PRINTS, ProDom, SMART, ProSite, and PANTHER databases). KEGG was used to analyze the functional protein families and pathways associated with the identified proteins. Volcano map analysis, cluster heatmap analysis and pathway enrichment analysis of GO, IPR and KEGG data were performed for the differentially expressed proteins and STRING software was used to predict possible PPIs (http://STRING.embl.de/) [20].

### Detection of 5'UTR translation initiation efficacy via a luciferase reporter assay

The RPS6 overexpression plasmid and its control plasmid, the MDM2 luc-5' UTR plasmid and its control plasmid were constructed by Genechem Company (Shanghai, China). 293 T cells seeded in 24-well plates were cotransfected with the above double plasmids together with a TK promoter-driven Renilla luciferase reporter plasmid. The cells were then collected and lysed 48 h after transfection, and the supernatant was obtained via centrifugation. Then, the activities of firefly and Renilla luciferases were calculated via a dual luciferase reporter assay system (Beyotime, RG029S). The relative luciferase activity of the MDM2 luc–5'UTR plasmid was further normalized to the signal in 293 T cells transfected with the firefly luciferase vector control under the same treatment conditions [21].

### MDM2 translation efficiency detection via a polysome profiling assay

Briefly, THP-1 cells transfected with shRPL6 or shCTRL lentivirus were treated with 100 µg/ml CHX (M4879, Abmole) for 10 min and collected. A sample of 3 × 10^7^ cells from each group was harvested, rinsed in cold PBS with 100 µg/ml CHX and quickly frozen in liquid nitrogen before lysis. The lysis buffer was composed of 10 mM Tris–HCl (pH 7.5), 100 mM NaCl, 30 mM MgCl2, and 100 µg/ml CHX with freshly added protease inhibitor (78,429, Thermo Fisher) and RNase inhibitor (R0102-2kU, Beyotime). The cytoplasm was subsequently extracted and layered onto a 10–50% sucrose gradient and centrifuged at 36,000 rpm for 2.5 h at 4 °C in an ultracentrifugation centrifuge (Beckman, XE-100). The sample was fractionated into 18 fractions (0.5 ml per fraction), and the RNA from 18 fractions was extracted and reverse-transcribed into cDNA. The abundance of MDM2 in 18 fractions was analyzed via RT-qPCR [21].

### Public database analysis

Processed single-cell RNA-seq data of BM mononuclear cells both before and after induction therapy, along with the corresponding annotation files, were downloaded from GSE116256 [12]. The annotation file provides information on the cell types corresponding to each cell, while the expression matrix presents the number of unique molecular identifiers for each gene (rows) across cells (columns). The post-chemotherapy LSC population was further categorized into subgroups based on the expression levels of a specific set of genes associated with cellular senescence. We utilized AML datasets from TCGA for survival analysis and to evaluate PSMB10 gene expression levels in patients with various FAB subtypes of AML. Public single-cell RNA-seq data (EGAD00001008373 and GSE185991) were obtained and subjected to unsupervised clustering analysis. The populations of LSCs and hematopoietic stem cells (HSCs) were identified based on multiple established molecular markers. We compared the expression levels of PSMB10 in both LSCs and HSCs, as well as the dynamic changes in PSMB10 levels at different stages of treatment within the same patient. Additionally, bulk RNA-seq data (GSE30029) were analyzed to assess PSMB10 gene expression levels in CD34^+^ bone marrow cells from patients with AML and healthy controls.

### Code availability

The code used for R analysis is available from the corresponding author upon reasonable request.

### Human leukemia cell-derived xenograft model

For human AML xenografts, 6- to 8-week-old female NCG mice were used for the ‘human-in-mouse’ AML xenograft model. For all the experiments below, the mice were randomly assigned to each group and the sample number was estimated based on extensive experience. Briefly, we transplanted 1 × 10^6^ shCTRL- and shPSMB10-transduced THP-1 cells into each sublethally irradiated (200 cGy) NCG mouse. For the shCTRL and shPSMB10 groups, THP-1 cells were suspended in 200 μl of PBS via tail vein injection. For the T-cell-treated group, 0.5 × 10^6^ activated human CD3^+^ T cells were intravenously injected immediately after the transplantation of 1 × 10^6^ shCTRL- or shPSMB10-transduced THP-1 cells [22]. For survival curve experiments, the deaths of the 1st transplantation recipient mice were recorded when the moribund animals were euthanized. The progression of leukemia was monitored once a week by testing the percentage of human CD45^+^ AML cells in PB after transplantation. The animals were euthanized at 5 weeks post-transplantation, and BM cells from the femurs and tibias, PB and spleen were collected from each mouse. The percentage of hCD45^+^ cells was analyzed with an anti-human APC-CD45 (304,012, Biolegend) antibody by FCM using a BD LSRFortessaTM X-20 special order product (Becton), and the size of the spleen was photographed. To further confirm the effect of PSMB10 on the maintenance of AML stemness, we performed secondary transplantation assays. BM cells from the same group of 1st transplantation recipient mice were mixed, and the percentage of hCD45^+^ cells was analyzed. Then, 1 × 10^6^ hCD45^+^ BM cells from the 1st transplantation recipient mouse were injected into another recipient mouse irradiated with 200 cGy via the tail vein. For the T-cell transplantation group, 0.5 × 10^6^ activated human CD3^+^ T cells per recipient mouse were intravenously injected. For survival curve experiments, the deaths of 2nd transplantation recipient mice were recorded when the moribund animals were euthanized. The animals were euthanized at 5 weeks post-transplantation, BM cells from the femurs and tibias, PB and spleen were collected from each mouse, and the percentage of hCD45^+^ cells was analyzed via FCM with an anti-human APC-CD45 (304,012, Biolegend) antibody. The sizes of the spleen and liver were determined. For limiting dilution analysis, 1 × 10^3^, 1 × 10^4^, 1 × 10^5^ and 1 × 10^6^ hCD45^+^ BM cells from the 1st transplantation recipient mice in the corresponding groups were transplanted into other sublethally (200 cGy) irradiated NCG mice. For the T-cell transplantation group, activated T cells were intravenously injected at an effector-to-target ratio of 1:2. The frequency of LSCs was analyzed via ELDA software (http://bioinf.wehi.edu.au/software/elda/).

### Syngeneic mouse bone marrow transplantation

BM cells from 8- to 10-week-old *Psmb10*^*−/−*^ and wild-type (WT) C57BL/6 J mice were harvested, and lineage-negative (Lin^−^) cells were isolated via a stem-progenitor cell sorting kit (19,856, StemCell Technologies). Then, the cells were infected with MLL-AF9-GFP retroviruses twice in IMDM with 8 µg/ml polybrene, 10 ng/ml recombinant mouse IL-3, 10 ng/ml recombinant mouse IL-6, and 20 ng/ml recombinant mouse SCF. For the first transplantation, 4 × 10^5^ infected MLL-AF9-GFP^+^ cells were injected into 8- to 10-week-old female sublethally (5.5 Gy) irradiated C57BL/6 J mice via the tail vein. On day 14 after transplantation, both *Psmb10* WT MLL-AF9 and *Psmb10*^*−/−*^ MLL-AF9 mice were randomly divided into two groups. For the treatment group, the mice were intraperitoneally injected with doxorubicin (25,316–40-9, MCE) at 5 mg/kg body weight for 3 consecutive days and with cytarabine (147–94-4, MCE) at 100 mg/kg body weight for 5 consecutive days every 24 h [23, 24]. For survival curves, the deaths of the 1st transplantation recipient mice were recorded when the moribund animals were euthanized. For secondary transplantation, 4 × 10^5^ GFP^+^ leukemia cells from the BM of 1st transplantation recipient leukemia mice, which were sacrificed on day 21 after the 1st transplantation, were injected into another sublethally irradiated 8- to 10-week-old female C57BL/6 J mouse via the tail vein. For the survival curve, the deaths of the 2nd transplantation recipient mice were recorded when the moribund animals were euthanized. When some mice showed signs of severe leukemia, all the mice were sacrificed at the same time. The leukemia burden was detected by determining the percentage of GFP^+^ leukemic cells in the spleen, PB, and BM via FCM. The size and weight of the spleen were also recorded. The percentage of LSCs and the CD93 intensities on LSCs in the BM were detected. Red blood cells (RBCs) were removed with RBC lysis buffer, and the remaining cells were stained with PerCp-Cy5.5 Mouse Lineage Antibody Cocktail (561,317, BD), PE-Cy7-anti-mouse CD127 (560,733, BD), BV605-anti-mouse c-Kit (752,696, BD), PE-anti-mouse CD16/32 (561,727, BD), BV421-anti-mouse CD34 (562,608, BD), APC-anti-mouse Sca-1 (17–5981-81, eBioscience), and BV510-anti-mouse CD93 (744,942, BD) antibodies. The cells were subsequently analyzed via FCM via a BD LSRFortessaTM X-20 special order product (Becton), and the data were analyzed via FlowJo software. The percentage of LSCs was defined as the percentage of GFP^+^Lin^−^Sca1^−^IL7R^−^c-Kit^+^CD16/32^high^CD34^+^ cells. The percentage of CD93 on LSCs was defined as the percentage of GFP^+^Lin^−^Sca1^−^IL7R^−^c-Kit^+^CD16/32^high^CD34^+^CD93^+^ cells.

### Hematopoietic function assay of *Psmb10*^*−/−*^ mice

Sixteen-week-old *Psmb10*^*−/−*^ and *Psmb10* WT C57BL/6 J mice were utilized to test hematopoietic function. First, the venous blood of the mice was collected for routine blood tests. Then, the mice were sacrificed, and the BM cells were harvested from the femur and tibia. RBCs were removed from the RBC lysis buffer, and the remaining cells were stained with PerCp-Cy5.5 Mouse Lineage Antibody Cocktail (561,317, BD), BV605-anti-mouse c-Kit (752,696, BD), PE-anti-mouse CD16/32 (561,727, BD), BV421-anti-mouse CD34 (562,608, BD), APC-anti-mouse Sca-1 (17–5981-81, eBioscience), APC/CY7-anti-mouse CD48 (561,242, BD), and PE/CY7-anti-mouse CD150 (115,913, Biolegend) antibodies. The cells were subsequently analyzed via FCM via a BD LSRFortessaTM X-20 special order product (Becton), and the data were analyzed via FlowJo software. The HSCs and different progenitor groups were defined as follows: HSCs: Lin^−^Sca1^+^c-Kit^+^CD48^−^CD150^+^; progenitor cells (Prog): Lin^−^Sca1^−^c-Kit^+^; common myeloid progenitors (CMPs): Lin^−^Sca1^−^c-Kit^+^CD34^+^CD16/32^−^; granulocyte–macrophage progenitors (GMPs): Lin^−^Sca1^−^c-Kit^+^CD34^+^CD16/32^+^; and megakaryocyte-erythroid progenitors (MEPs): Lin^−^Sca1^−^c-Kit^+^CD34^−^CD16/32^−^.

### Ethical approval declarations

The bone marrow cells from AML patients and healthy donors used in the experiment were obtained from Wuhan Union Hospital, and informed consent was obtained from the patients. The procedures were approved by the Ethics Committee of Huazhong University of Science and Technology Tongji Medical College, and the methodologies involved comply with relevant operational guidelines. The mice were raised in an SPF-level environment and provided with adequate humane care. All experimental procedures involving the mice complied with applicable regulations and were approved and supported by the Ethics Committee of Huazhong University of Science and Technology.

### Statistical analysis

Data analysis was performed with GraphPad Prism 8, and the data are presented as the means ± SDs. Statistical significance was calculated via unpaired Student’s t test. Kaplan‒Meier survival curves were plotted with GraphPad Prism 8, and the log-rank test was used to compare the significance. All experiments were independently performed at least three times, the samples of AML cell lines, AML patients, and mice for each experiment were no less than three. In our figures, asterisks indicate *p < 0.05, **p < 0.01, ***p < 0.001, and ****p < 0.0001.

## Results

### The specific increased expression of PSMB10 in post-chemotherapy nonsenescent LSCs predicts poor AML prognosis

**Fig. 1:**
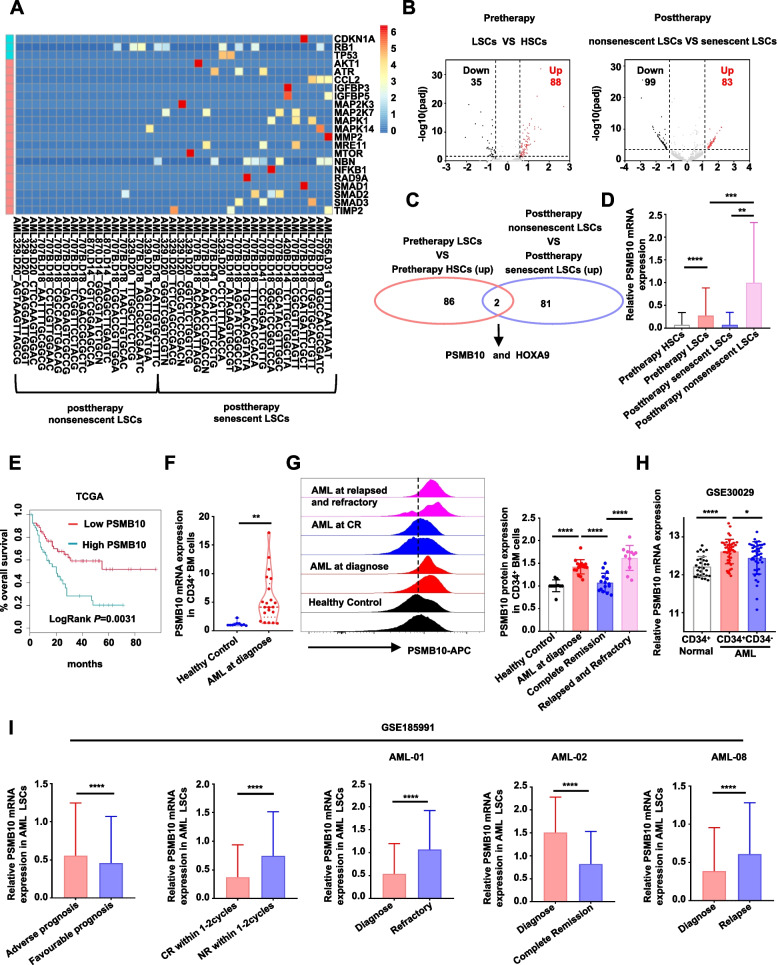
The specific increased expression of PSMB10 in post-chemotherapy nonsenescent LSCs predicts a poor AML prognosis. **A** Single-cell RNA-seq data (GSE116256) showing postchemotherapy LSC clusters defined by senescence-associated genes.** B** Volcano plot showing differentially expressed genes of the defined cell population. **C** The overlapping upregulated genes between pretherapy LSCs and post-therapy nonsenescent LSCs.** D** PSMB10 mRNA expression in defined cell populations. **E** Kaplan‒Meier plots of the overall survival of AML patients in TCGA. **F** PSMB10 mRNA levels in BM CD34^+^ cells from healthy donors (*n* = 10) and primary AML patients (*n* = 21) were determined via RT-qPCR. **G** Relative PSMB10 protein levels in BM CD34^+^ subsets from healthy individuals (*n* = 10), primary AML patients (*n* = 17), AML patients with complete remission (*n* = 15), and relapsed/refractory patients (*n* = 11) analyzed via FCM. **H** PSMB10 mRNA levels in normal CD34^+^ cells, AML CD34^+^ cells, and AML CD34^−^ cells from GSE30029. **I** PSMB10 mRNA levels in AML LSCs with different prognoses (left 1st), therapeutic responses (left 2nd), and therapeutic periods (right three) from single-cell RNA-seq data (GSE185991). LSCs: leukemia stem cells; HSCs: hematopoietic stem cells; CR: Complete Remission; NR: No Response. *p < 0.05, **p < 0.01, ***p < 0.001, and ****p < 0.0001 (t test). The error bars denote the means ± SDs

To identify the key molecules responsible for senescence resistance in drug-resistant LSCs, we analyzed an available single-cell RNA-seq dataset [12]. Our findings indicated that the post-chemotherapy residual LSCs could be categorized into nonsenescent and senescent clusters (Fig. 1A). Differential gene expression analysis (fold change ≥ 2.5, *p* < 0.01) revealed that 88 genes were significantly upregulated in pre-therapy LSCs compared to pre-therapy HSCs, while 83 genes showed significant upregulation in post-therapy nonsenescent LSCs relative to post-therapy senescent LSCs (Fig. 1B). We intersected the two sets of upregulated differentially expressed genes and identified two overlapping genes, *PSMB10* and *HOXA9* (Fig. 1C). It has been reported that *HOXA9* is implicated in the resistance to AML1-ETO-induced senescence in AML cells by suppressing *p16*^*Ink4a*^ expression [25]. However, it remains to be confirmed whether the *PSMB10* also contributes to senescence resistance in these chemotherapy-resistant non-senescent LSCs.

The further question is whether the increased *HOXA9* and *PSMB10* lead to senescence resistance in these LSCs in an independent or interdependent manner. Our analysis of the published data (GSE13714) revealed that the knockdown of *HOXA9* in AML cells did not affect their PSMB10 mRNA expression levels [26]. Furthermore, HOXA9 does not function as a transcriptional regulator of *PSMB10* [27], indicating that the increased HOXA9 doesn’t lead to an upregulated PSMB10. Besides, there is no evidence that HOXA9 results in increased PSMB10 activity by molecular modification or change in structure yet. Therefore, the increased HOXA9 could not induce resistance to senescence in LSCs via the upregulated PSMB10, and they could independently play significant roles in maintaining stemness in drug-resistant non-senescent LSCs.

In addition, HOXA9 is a transcription factor with complex downstream regulatory networks that remain to be fully elucidated [28, 29]. Furthermore, there is a so-called "undruggable" characteristic inherent to transcription factors, making it difficult to directly target with small-molecule drugs [30]. As a result, current research has to shift towards finding its upstream regulators or downstream target genes as alternative targets. In contrast, as an immunoproteasome subunit, PSMB10 has well-defined structural and functional characteristics, which provide an established framework for developing small-molecule inhibitors or modulators. This inherent advantage in drug-targetability makes PSMB10 a promising candidate for clinical translational research. Thus, the increased PSMB10 was selected as a target molecule for our further analyses.

The following analysis revealed that the expression of *PSMB10* was significantly upregulated in post-chemotherapy nonsenescent LSCs (Fig. 1D), with a 13-fold increase (*p* = 0.0016) relative to post-chemotherapy senescent LSCs. By analyzing an AML cohort from The Cancer Genome Atlas (TCGA), we found that AML patients with higher *PSMB10* expression had significantly poorer overall survival (Fig. 1E). Our RT-qPCR analysis demonstrated that PSMB10 mRNA expression in CD34^+^ BM cells from AML patients was considerably higher than that in cells from healthy controls (Fig. 1F). Additionally, our flow cytometry (FCM) results revealed that there was an increased PSMB10 protein expression in CD34^+^ cells from AML patients, particularly in those who were relapsed or refractory, compared to healthy controls. Notably, PSMB10 expression was downregulated in patients who achieved complete remission (CR) (Fig. 1G). The bulk RNA-seq data revealed that *PSMB10* expression was higher in the BM CD34^+^ cells of AML patients compared to both BM CD34^−^ AML cells and BM CD34^+^ cells from healthy donors (Fig. 1H). By analyzing single-cell RNA-seq data with a panel of stemness genes to define LSCs [31] (Supplementary Table 3), we found that LSCs have higher *PSMB10* expression than HSCs do in AML patients (Supplementary Fig. S1A and S1B left). Patients with adverse prognoses had higher levels of PSMB10 expression in LSCs than those with favorable prognoses did, and patients with no response (NR) after 1–2 treatment cycles had higher levels of *PSMB10* expression in LSCs than those who achieved CR did (Fig. 1I left two). Notably, *PSMB10* expression increased in LSCs from diagnosis to relapse and refractory but downregulated from diagnosis to CR (Fig. 1I right three, Supplementary Fig. S1B right). The FAB classification is a widely used system for categorizing AML based on the morphological and cytochemical characteristics of leukemic cells. It divides AML into subtypes M0 to M7, each representing distinct stages of myeloid differentiation and specific pathological features [32, 33]. The TCGA database indicated that PSMB10 expression levels vary among different AML FAB subtypes: M5 exhibits the highest expression, while M3 shows the lowest (Supplementary Fig. S1C), indicating that *PSMB10* may play a significant role in non-M3 AML, particularly in M5 AML. Overall, these data suggest that increased *PSMB10* expression in LSCs may be a key driver of relapsed and refractory AML and is associated with poor prognosis in AML patients.

### Downregulation of *PSMB10* restarts senescence and promotes CTL-mediated killing of AML cells in vitro

**Fig. 2:**
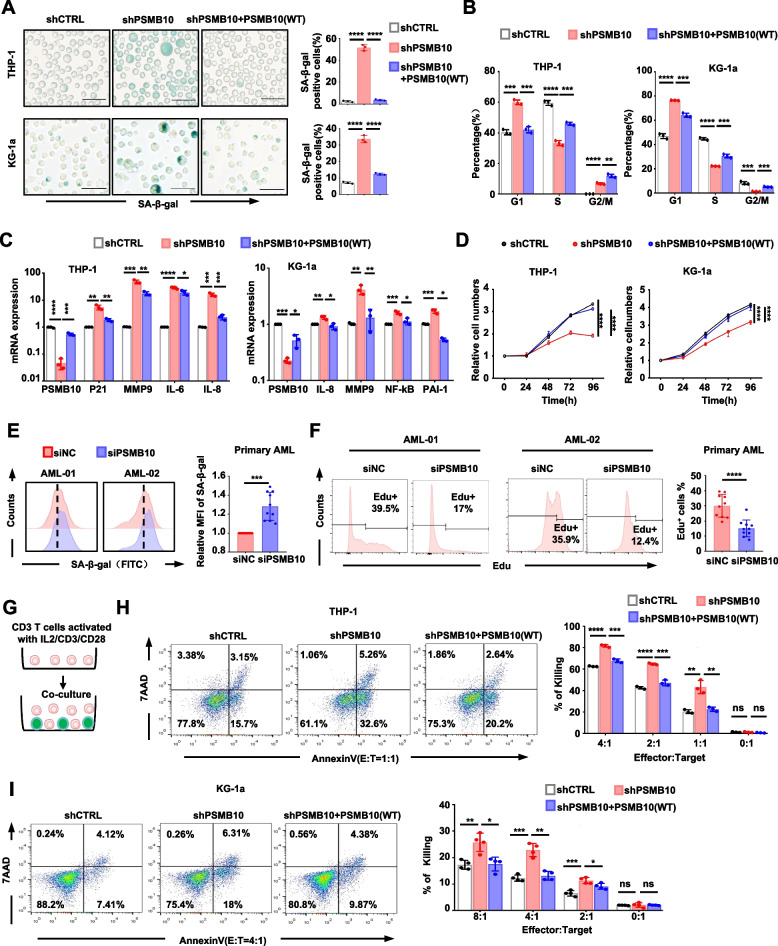
Downregulation of PSMB10 restarts senescence and promotes CTL-mediated killing of AML cells in vitro. **A-D** Representative images of SA-β-Gal staining (left) and the number of SA-β-Gal^+^ cells (right) (**A**), cell cycle distribution (**B**), PSMB10 and SASP mRNA expression (**C**), and growth curves (**D**) of THP-1 and KG-1a cells after transduction with the indicated lentiviruses. Scale bar, 50 μm. **E–F** Statistical histogram of SA-β-gal (**E**) and EdU^+^ percentage (**F**) for AML patient-derived BM CD34^+^ cells (FAB: M0/M1/M2, *n* = 5) or BM mononuclear cells (FAB: M5, *n* = 5) after PSMB10 downregulation with the indicated siRNA.** G** Scheme for a co-culture system of T cells and AML cells. Created with figdraw.com. **H-I** Percentage of Annexin V^+^ apoptotic THP-1 (**H**) and KG-1a (**I**) cells after coculture with activated CD3^+^ T cells. SA-β-Gal: senescence-associated β-galactosidase; WT: wild-type; siNC: small interfering RNA negative control; MFI: mean fluorescence intensity. *p < 0.05, **p < 0.01, ***p < 0.001, and ****p < 0.0001 (t test). The error bars denote the means ± SDs

To investigate the role of *PSMB10* in leukemia maintenance, we first used short hairpin RNA (shRNA) to KD *PSMB10* expression (shPSMB10) in two human AML cell lines (THP-1 and KG-1a). Subsequently, we restored *PSMB10* expression by inserting its cDNA into the cells that were previously transfected with shPSMB10 (Supplementary Fig. S2A). We found that PSMB10 KD increased senescence-associated β-galactosidase (SA-β-Gal) activity (Fig. 2A), induced cell cycle arrest at G_O_/G_1_ (Fig. 2B, Supplementary Fig. S2B), upregulated senescence-associated secretory phenotype (SASP) factors (Fig. 2C), and significantly suppressed cell growth (Fig. 2D, Supplementary Fig. S2C). All these biological changes were successfully reversed by wild-type (WT) *PSMB10*. To further verify the function of *PSMB10* in primary AML patients, we utilized *PSMB10* small interfering RNA (siRNA) to reduce its expression (siPSMB10) in CD34^+^ BM cells (FAB, M0/M1/M2) and BM mononuclear cells (FAB, M5) isolated from AML patients, we similarly found that *PSMB10* KD increased SA-β-Gal activity (Fig. 2E) and reduced the EdU^+^ percentage (Fig. 2F) in these primary AML cells. Besides, the change in PSMB10 levels in THP-1 and KG-1a cells did not affect their degree of apoptosis (Supplementary Fig. S2D). These results suggest that the downregulation of *PSMB10* in AML cells can induce senescence without causing apoptosis.

To further determine whether *PSMB10* maintained the stemness of leukemia cells via immune escape, we cocultured THP-1 or KG-1a cells transfected with the indicated lentivirus and activated healthy human CD3^+^ T cells for 18 h (Fig. 2G) at different effector/target ratios (E: T) [19]. We found that *PSMB10* KD significantly sensitized AML cells to CTL killing, and this cytotoxicity was effectively rescued upon restoration of *PSMB10* expression (Fig. 2H and I), suggesting that *PSMB10* promotes the escape of leukemia cells from CTL-mediated killing.

### Downregulating *PSMB10* synergistically promotes LSC eradication in conjunction with T cells in vivo

**Fig. 3:**
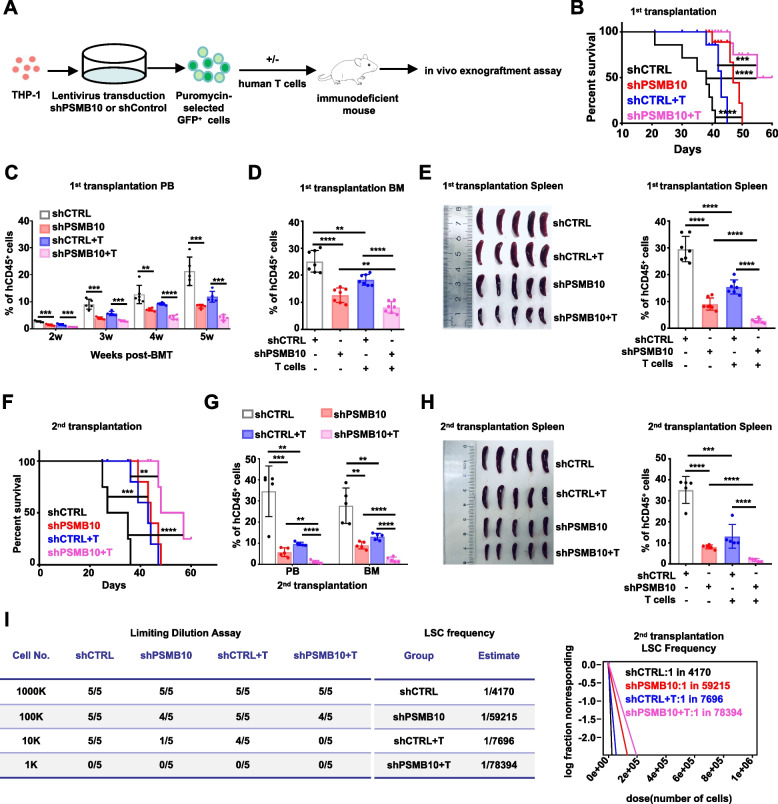
Downregulating PSMB10 synergistically promotes LSC eradication in conjunction with T cells in vivo. **A** Experimental Scheme for NCG mice transplantation. Created with figdraw.com. **B** Kaplan‒Meier survival plot of the 1st recipient NCG mice transplanted with shCTRL or shPSMB10 THP-1 cells or T cells (E: T = 1:2) (*n* = 8). **C** Percentages of human CD45^+^ leukemia cells in the PB at indicated times (*n* = 5–6). **D** Percentages of human CD45^+^ leukemia cells in the BM after the death of the 1st transplantation NCG mice at 5 weeks (*n* = 7).** E** Spleen size and percentage of hCD45^+^ leukemia cells in the spleen of the 1st transplantation recipient NCG mice at 5 weeks (*n* = 7). **F** Kaplan‒Meier survival plot of the 2nd transplantation recipient NCG mice (E: T = 1:2, *n* = 8).** G** Percentage of human CD45^+^ leukemia cells in the PB and BM after the death of the 2nd transplantation recipient NCG mice at 5 weeks (*n* = 5).** H** Spleen size and percentage of human CD45^+^ leukemia cells in the spleen of the 2nd transplantation recipient NCG mice after the death at 5 weeks (*n* = 5). **I** Limiting dilution assays. The table (left) shows different numbers and treatment methods of transplanted leukemic cells with or without T cells and death numbers for each 2nd transplantation recipient NCG mouse. The table (middle) shows the LSC frequency in each transplantation recipient NCG mouse group. Limiting dilution assays of the 2nd transplantation showing the frequency of LSCs (right). BM: bone marrow; PB: peripheral blood; GFP: green fluorescent protein. **p < 0.01, ***p < 0.001, and ****p < 0.0001 (t test). The error bars denote the means ± SDs

To assess the effect of downregulating *PSMB10* on AML progression in vivo, we employed an AML xenograft model with NOD/ShiLtJGpt-*Prkdc*^em26Cd52^*Il2rg*^em26Cd22^/Gpt (NCG) mice (Fig. 3A). The shPSMB10 THP-1 cells + T cells co-transplant group, related to control groups, had markedly prolonged survival in the 1st transplantation NCG mice (shPSMB10 + T cells: 58 days vs shCTRL cells: 38 days, *p* < 0.0001; Fig. 3B) and a lower percentage of human CD45^+^ (hCD45^+^) leukemia cells in the peripheral blood (PB), BM, and spleen and less severe splenomegaly (Fig. 3C-E) when the mice were sacrificed at 5 weeks. After transplanting 1 × 10^6^ hCD45^+^ leukemia cells from the 1st bone marrow transplantation (BMT) recipient mice without or with 0.5 × 10^6^ activated T cells in the corresponding groups, into another NCG mouse, we found that compared to other control groups, the shPSMB10 THP-1 cells + T cells co-transplant group had significantly prolonged survival (shPSMB10 + T cells: 53 days vs shCTRL cells: 31 days, *p* < 0.0001; Fig. 3F), a substantially reduced leukemia burden in the PB, BM, and spleen (Fig. 3G and H), and a 19-fold decrease in the frequency of LSCs related to that in shCTRL THP-1 cells transplant group (1/78394 vs 1/4170, *p* = 0.000183; Fig. 3I) in the 2nd transplantation NCG mice.

### Loss of *PSMB10* boosts chemotherapy-induced senescence in vitro and eradication of drug-resistant LSCs in vivo

**Fig. 4:**
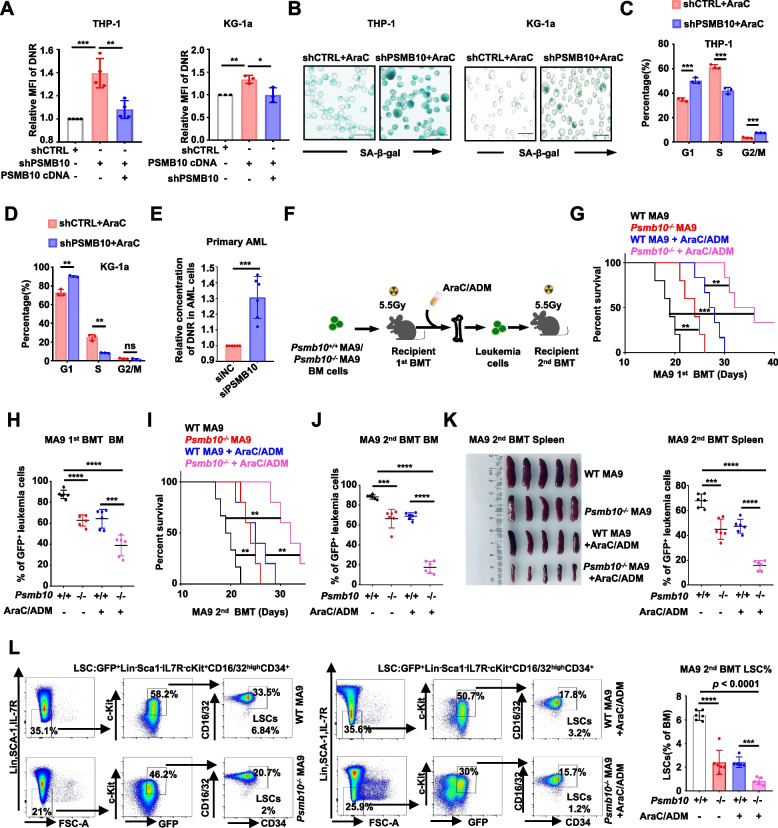
Loss of PSMB10 boosts chemotherapy-induced senescence in vitro and eradication of drug-resistant LSCs in vivo.** A** Statistical histogram of the mean fluorescence intensity of daunorubicin in the indicated lentivirus-transfected THP-1 and KG-1a cells. **B-D** Representative images of SA-β-gal staining (**B**) and the cell cycle distribution (**C-D**) in THP-1 and KG-1a cells transduced with the indicated lentiviruses under AraC (0.2 ug/ml) treatment. Scale bar, 50 µm. **E** Statistical histogram of the mean fluorescence intensity of daunorubicin in AML patient-derived BM CD34^+^ cells (FAB: M0/M1/M2) or BM mononuclear cells (FAB: M5) after transduction with the indicated siRNAs. **F** Experimental scheme for **G-L**. Created with figdraw.com. **G** Kaplan–Meier survival curves of C57BL/6J mice transplanted with either *Psmb10*^+*/*+^ or *Psmb10*^*−/−*^ MLL-AF9 leukemic cells, as well as those treated with chemotherapy drugs via intraperitoneal injection on day 14 post 1st BMT (*n* = 8). **H** Percentages of GFP^+^ leukemia cells in the BM after the death of 1st BMT recipient C57BL/6 J mice on day 21 (*n* = 6). **I** Kaplan‒Meier survival curves for 2nd BMT recipient C57BL/6 J mice (*n* = 8). **J-K** Percentages of GFP^+^ leukemia cells in BM (**J**) and spleen (**K**), as well as spleen size after the death of 2nd BMT recipient C57BL/6 J mice (*n* = 6). **L** Representative FCM images and statistical histograms illustrating the percentage of LSCs after the death of 2nd BMT recipient C57BL/6 J mice at 4 weeks (*n* = 5–6). SA-β-Gal: senescence-associated β-galactosidase; siNC: small interfering RNA negative control; BM: bone marrow; AraC: Arabinoside Cytosine; DNR: Daunorubicin; ADM: Adriamycin; MA9: MLL-AF9; BMT: bone marrow transplantation. *p < 0.05, **p < 0.01, ***p < 0.001 and ****p < 0.0001 (t test). ns, not significant. The error bars denote the means ± SDs

We found that *PSMB10* KD increased the intracellular mean fluorescence intensity (MFI) of DNR in both THP-1 and KG-1a cells (Fig. 4A). As we used AraC to induce leukemia senescence, we discovered that the downregulation of *PSMB10* increased SA-β-Gal activity (Fig. 4B), the proportion of cells in G_0_/G_1_ phase (Fig. 4C and D) and SASP factor levels, and decreased cell cycle-associated gene expression (Supplementary Fig. S3A) in different AML cell lines. As shown in the Supplementary Fig. S3B, the PSMB10 knockdown combined with AraC treatment leads to an increased P21 protein expression, accompanied by significant reductions in CDK2, CDK4, and CDK6, as well as cyclin E2 (CCNE2) protein expressions. Importantly, we also observed markedly reduced phosphorylation of Rb at Ser807/811 sites, which serves as the direct evidence of diminished CDK kinase activity [34]. After employing siRNA to reduce *PSMB10* expression in primary CD34^+^ BM cells (FAB, M0/M1/M2) and BM mononuclear cells (FAB, M5) isolated from AML patients, we found that *PSMB10* downregulation similarly increased the intracellular DNR concentration in these primary AML cells (Fig. 4E).

To further investigate whether *PSMB10* downregulation could increase primary LSC sensitivity to chemotherapy in vivo with syngeneic normal hematopoietic and immune microenvironment, we used the MLL-AF9 (MA9) leukemia mouse model (Fig. 4F). By testing the percentage of GFP^+^ leukemia cells in the PB of mice at 1–2 weeks after 1st transplantation, we found that *Psmb10* depletion reduced the leukemia burden in the PB of these mice (Supplementary Fig. S3C). Then, drug treatment was performed 14 days after the 1st transplantation, as previously reported [23, 24]. Mice receiving *Psmb10*^−/−^ MA9 bone marrow cells as the 1st recipients, combined with chemotherapy, exhibited longer survival compared to those receiving *Psmb10*^+/+^ MA9 cells **(**34 days vs 19 days, *p* = 0.0007; Fig. 4G), and the *Psmb10*^−/−^ MA9 BM cells 1st recipient mice combined with chemotherapy had lower numbers of GFP^+^ leukemia cells in the BM, PB and spleen and less severe splenomegaly than did the *Psmb10*^+/+^ MA9 1st recipient mice (Fig. 4H, and Supplementary Fig. S3D and E). A significant delay in AML development and a reduction in mortality were observed in the 2nd recipient mice transplanted with AraC- and doxorubicin (ADM)-treated *Psmb10*^−/−^ MA9 mouse BM leukemia cells, compared to those receiving *Psmb10*^+/+^ MA9 cells **(**32 days vs 19.5 days, *p* = 0.0014; F ig. 4I). Additionally, a marked decrease in the percentage of GFP^+^ leukemic cells was noted in the BM, spleen, and PB of the 2nd recipient mice transplanted with AraC- and ADM-treated *Psmb10*^−/−^ MA9 mouse BM leukemia cells, compared to those receiving *Psmb10*^+/+^ MA9 cells (Fig. 4J and K, Supplementary Fig. S3F). We also found significantly decreased LSC populations (0.8428 ± 0.3184% vs 6.38 ± 0.41%; *p* < 0.0001; Fig. 4L) along with a reduction in CD93^+^ (positively correlated with leukemia stemness [35]) LSCs (14.1 ± 2.85% vs 27.13 ± 5.06%; *p* = 0.0007; Supplementary Fig. S3G) in the BM of the 2nd receptor model mice transplanted with AraC- and ADM-treated *Psmb10*^−/−^ MA9 mouse BM cells, compared to those receiving *Psmb10*^+/+^ MA9 cells. These results indicate that the downregulation of *PSMB10* can enhance drug-induced cellular senescence and promote the clearance of drug-resistant LSCs in vivo.

### The increased PSMB10 impedes RPL6/RPS6-MDM2-P21-induced senescence initiation in AML cells

**Fig. 5:**
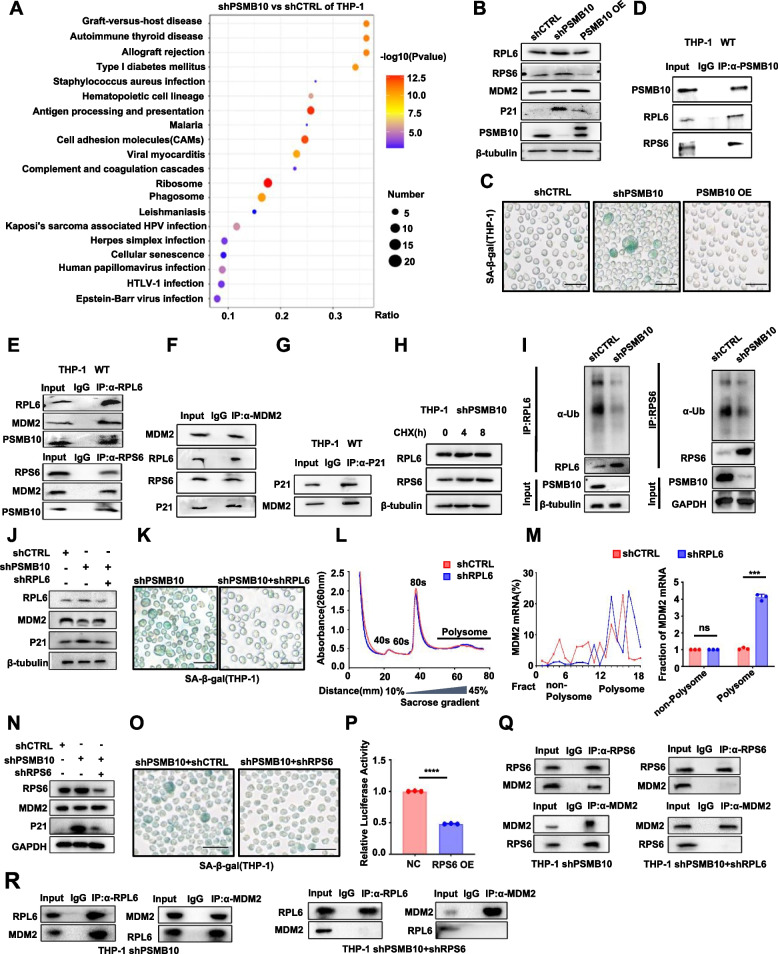
The increased PSMB10 impedes RPL6/RPS6-MDM2-P21-induced senescence initiation in AML cells.** A** Scattergram of upregulated pathways based on KEGG analysis of quantitative proteomics. **B-C** Changes in protein levels (**B**) and representative images of SA-β-Gal staining (**C**) in the indicated lentivirus-transfected THP-1 cells. Scale bar, 50 μm. **D-G** Immunoprecipitation assay between RPL6 or RPS6 and PSMB10 (**D**), RPS6 (top) or RPL6 (bottom) and MDM2 or PSMB10 (**E**), MDM2 and RPL6 or RPS6 or P21 (**F**), and MDM2 and P21 (**G**) in THP-1 cells. **H** shPSMB10-transfected THP-1 cells were treated with CHX for the indicated times, and RPS6 and RPL6 protein levels were detected via WB analysis.** I** Ubiquitination assay of (left) RPL6 and (right) RPS6 in shPSMB10- or shCTRL-transfected THP-1 cells. **J-K** Changes in the protein levels of RPL6, MDM2, and P21 (**J**), and representative images of SA-β-Gal staining (**K**) in shCTRL- and shRPL6-transduced THP-1 cells with shPSMB10. Scale bar, 50 μm. **L** Polysome profiling of shRPL6- or shCTRL-transduced THP-1 cells. **M** Polysome profiling coupled with qRT‒PCR analysis of shCTRL- and shRPL6-transduced THP-1 cells: MDM2 mRNA distribution in different ribosome fractions (left), and statistical histogram of MDM2 mRNA in the nonribosome portion and polysomes (right). **N–O** Changes in the protein levels of RPS6, MDM2, and P21 (**N**), and representative images of SA-β-Gal staining (**O**) in shCTRL- and shRPS6-transduced THP-1 cells with shPSMB10. Scale bar, 50 μm. **P** Statistical histogram of MDM2 translation initiation efficacy, defined as the quotient of reporter protein production (F-luc/R-luc). **Q** Immunoprecipitation assay between RPS6 and MDM2 in control shRNA- or RPL6 shRNA-transfected THP-1 cells transfected with shPSMB10. **R** Immunoprecipitation assay between RPL6 and MDM2 in control shRNA- or RPS6 shRNA-transfected THP-1 cells transfected with shPSMB10. OE: overexpression; SA-β-Gal: senescence-associated β-galactosidase; WT: wild-type; IP: immunoprecipitation; CHX: cycloheximide; Fract: fraction; IgG: Immunoglobulin G; NC: negative control; Ub: ubiquitination. ****p < 0.0001 (t test). ns, not significant. The error bars denote the means ± SDs

To decipher the mechanism by which increased *PSMB10* promotes leukemia cell resistance to senescence, THP-1 cells were transfected with shPSMB10, PSMB10-overexpressing (PSMB10-OE) or control (shCTRL) lentivirus. Our quantitative proteomic revealed that the activated ribosome pathway [especially ribosomal protein (RP) L6 and RPS6] was the top activated signaling pathway in the shPSMB10 group compared to the shCTRL group (Fig. 5A). Based on these results combined with our previous discovery that *P21* expression was increased coincided with a reduction in cell cycle regulators in the siPSMB10 group relative to the siNC group (Supplementary Fig. S4A and B), we constructed a protein–protein interaction (PPI) network of senescence-initiating molecular signaling pathways based on STRING database (https://string-db.org/) [20] (Supplementary Fig. S4C), suggesting that downregulated PSMB10 could induce senescence through the RPL6/RPS6-MDM2-P21 pathway. Our western blot (WB) results confirmed the increased levels of the RPL6, RPS6, and P21 but decreased MDM2 levels in the shPSMB10 group relative to those in the shCTRL and PSMB10-OE groups (Fig. 5B). We also discovered that the downregulation of PSMB10 increased SA-β-Gal activity in THP-1 cells (Fig. 5C). To further reveal the PPIs among the molecules of the signaling axis, we performed a coimmunoprecipitation (co-IP) assay of WT THP-1 cells. The results revealed interactions between PSMB10 and RPL6 or RPS6, between RPL6 or RPS6 and MDM2, and between MDM2 and P21 (Fig. 5D-G). To further clarify the mechanism by which PSMB10 downregulates RPL6 and RPS6, we determined the levels of the RPL6 and RPS6 proteins in PSMB10-KD THP-1 cells after exposure to the protein synthesis inhibitor cycloheximide (CHX) at the indicated times. WB analysis showed that the protein levels of both RPL6 and RPS6 remained unchanged (Fig. 5H), suggesting that PSMB10 downregulates the RPL6 and RPS6 proteins not via protein synthesis but through protein degradation. Next, we performed a ubiquitination assay. As shown in Fig. 5I and Supplementary Fig. S4D, shPSMB10 transduction strongly rescued RPL6 and RPS6 ubiquitination, suggesting that PSMB10 downregulated the RPL6 and RPS6 proteins via ubiquitination-mediated degradation.

To further investigate the role of RPL6 in regulating the senescence of AML cells, we first used shRNA to abrogate its expression in shPSMB10 THP-1 cells. RPL6 KD increased MDM2 but decreased P21 expression (Fig. 5J) without inducing cell senescence (Fig. 5K). Our RT-qPCR analysis indicated no significant difference in the mRNA expression of MDM2 among the shCTRL, shPSMB10, and shRPL6 groups (Supplementary Fig. S4E), suggesting that the increased MDM2 protein levels are not regulated through RPL6-mediated mRNA transcription. Because RPL6, an essential component of the large subunit (60S), is involved mainly in protein translation [36], we thus performed polysome profiling [21] in shRPL6- and shCTRL-transduced THP-1 cells. Our results revealed that there was no difference in total translational activity between the shCTRL and shRPL6 groups (Fig. 5L), however, we observed a notable increase in MDM2 mRNA within translation-active polysomes (> 80S) of the shRPL6-transduced THP-1 cells compared with the shCTRL cells (Fig. 5M), indicating that downregulation of RPL6 promoted the translation efficacy of MDM2.

RPS6 was found to be upregulated, similar to RPL6, in shPSMB10 THP-1 cells, and its KD also resulted in increased MDM2 and decreased P21 protein expression (Fig. 5N). RPS6 downregulation rescued senescence induction in the shPSMB10-transfected THP-1 cells (Fig. 5O). However, the downregulation of RPS6 did not affect the mRNA level of MDM2 (Supplementary Fig. S4F). Since RPS6 may play an important role in translational initiation [36], we constructed a pmirGLO-MDM2 luciferase reporter by ligating the MDM2-5'UTR coding region to the multiple cloning site (MCS) site (Supplementary Fig. S4G). The dual-luciferase assay revealed that the translation efficacy of MDM2 was significantly lower in the RPS6-overexpressing plasmid-transduced 293 T cells than in the control cells (Fig. 5P), indicating that RPS6 inhibits translation initiation of the MDM2 protein. It has been confirmed that MDM2 could promote the ubiquitin-independent degradation of P21^Waf1^ protein by inducing a change in its conformation [37–39]. These suggest that the PSMB10-mediated ubiquitinated degradation of both RPL6 and RPS6 results in increased MDM2 protein via the upregulation of translation activity, which further leads to degradation of P21^Waf1^ protein and resistance to senescence in AML cells.

Since we discovered that RPL6 and RPS6 had a direct molecular association with MDM2 separately (Fig. 5E and F), we further asked whether and how both RPL6 and RPS6 regulate MDM2 activity by their direct binding. After we performed a co-IP assay in shPSMB10- and shPSMB10 + shRPL6-transfected THP-1 cells, it was found that RPS6 cannot interact with MDM2 without RPL6 (Fig. 5Q). Similarly, as shown in Fig. 5R, RPS6 KD in shPSMB10 THP-1 cells led to no interaction between RPL6 and MDM2. These findings indicated that both RPL6 and RPS6 are essential for their mutual binding to MDM2. Previous studies have shown that the binding of RPs to MDM2 can induce its conformational change, which inhibits the interaction between p21 and MDM2, thereby impairing the ubiquitin-independent degradation of P21 protein [37–39]. These suggest that both RPL6 and RPS6 could directly bind to MDM2 as a protein complex to inhibit the MDM2-mediated degradation of P21 protein in AML cells. Thus, PSMB10-mediated ubiquitinated degradation of RPL6/RPS6 will result in decreased RPs binding-induced conformational change of MDM2, which will further promote MDM2-mediated degradation of P21 protein and resistance to senescence in AML cells.

### The increased PSMB10 impedes the SLC22A16-mediated drug endocytosis and senescent induction in AML cells

To further investigate whether and how *PSMB10* prevents AML cells from drug-mediated senescent induction via regulation of the intracellular drug concentration, we assessed DNR influx and efflux kinetics in shCTRL- and shPSMB10-transduced THP-1 cells [18]. The influx of daunorubicin (DNR) in shPSMB10-transfected THP-1 cells was significantly greater than that observed in shCTRL-transfected THP-1 cells after a 1-h influx period (Supplementary Fig. S4H and I), but that there was no difference in intracellular daunorubicin efflux (Supplementary Fig. S4J) during the efflux period between the shCTRL-transduced and shPSMB10-transduced THP-1 cells.

The organic cation transporter SLC22A16 has been shown to participate in the uptake of anthracyclines in leukemic cells [40, 41]. We found that SLC22A16 protein levels were higher in shPSMB10-transfected THP-1 cells than in shCTRL-transfected THP-1 cells (Supplementary Fig. S4K). Thus, PSMB10 may reduce intracellular drug concentrations by inhibiting SLC22A16-mediated endocytosis. To confirm this hypothesis, we performed a co-IP assay in THP-1 cells, and the results revealed that PSMB10 has a molecular interaction with SLC22A16 (Supplementary Fig. S4L). To further investigate how PSMB10 downregulates SLC22A16, we exposed shPSMB10-transfected THP-1 cells to CHX for the indicated times and found that the SLC22A16 protein level remained unchanged (Supplementary Fig. S4M). Additionally, we found that SLC22A16 ubiquitination was lower in shPSMB10-transfected THP-1 cells than in shCTRL-transfected THP-1 cells (Supplementary Fig. S4N), indicating that PSMB10 downregulates SLC22A16 protein expression in a ubiquitin-dependent manner. Thus, we conclude that PSMB10 decreases the intracellular drug concentration in leukemia cells by inhibiting SLC22A16-mediated drug endocytosis.

### Increased PSMB10 leads leukemia cell resistance to CTL-mediated killing through ubiquitinated degradation of MHC-I proteins

**Fig. 6:**
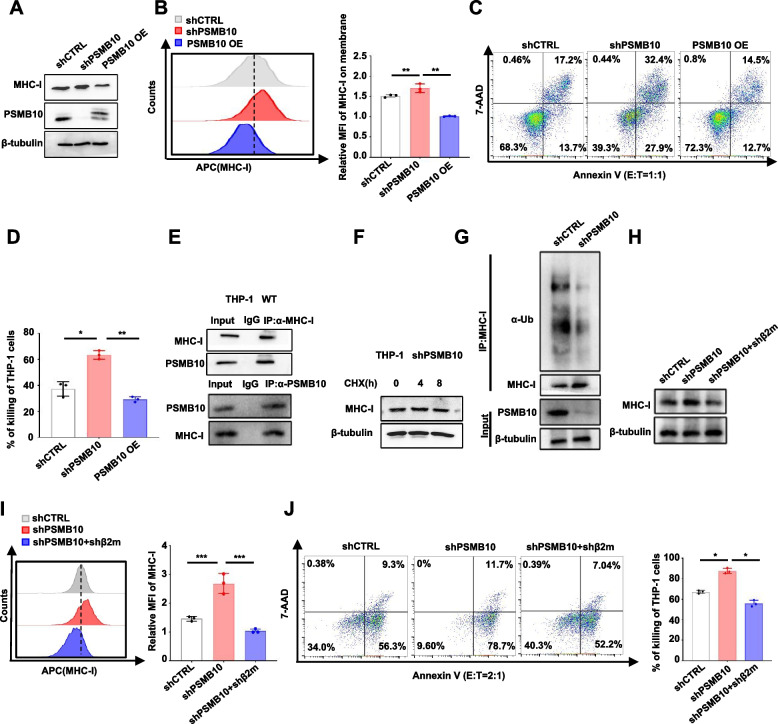
Increased PSMB10 leads leukemia cell resistance to CTL-mediated killing through ubiquitinated degradation of MHC-I proteins. **A-B** MHC-I protein level changes (**A**), representative FCM images, and statistical histogram (**B**) of membrane MHC-I protein levels. **C-D** Representative FCM images for the percentage of Annexin V^+^ apoptotic cells (**C**) and statistical histogram for the percentage of Annexin V^+^ apoptotic cells (**D**) in the indicated lentivirus-transfected THP-1 cells after co-culture with activated human CD3^+^ T cells for 18 h at an effector-to-target (E: T) ratio of 1: 1. **E** Immunoprecipitation assay of MHC-I and PSMB10 in THP-1 cells. **F** shPSMB10 cells were treated with CHX at the indicated times, and MHC-I protein levels were detected via WB analysis. **G** Ubiquitination assays of MHC-I in lysates from shCTRL- or shPSMB10-transduced THP-1 cells. **H** Representative WB images of total MHC-I protein in shCTRL- or shβ2m-transduced THP-1 cells transfected with shPSMB10. **I** Representative FCM images and statistical histogram of membrane MHC-I protein levels. **J** Representative FCM images and statistical histogram of the percentage of Annexin V^+^ apoptotic cells in shCTRL-, shPSMB10- and shβ2m-transduced THP-1 cells after co-culture with activated human CD3^+^ T cells for 18 h at an effector-to-target (E: T) ratio of 2: 1. MHC-I: major histocompatibility complex class I; CHX: cycloheximide; E: T: effector/target ratios; MFI: mean fluorescence intensity; OE: overexpression; IgG: Immunoglobulin G; Ub: ubiquitination. *p < 0.05, **p < 0.01, ***p < 0.001 and ****p < 0.0001 (t test). The error bars denote the means ± SDs

Our proteomics results revealed that downregulation of PSMB10 significantly increased the expression of genes enriched in the “antigen processing and presentation” pathway and the number of MHC-I molecules (Fig. 5A). WB and FCM analyses revealed increased MHC-I molecules in shPSMB10 THP-1 cells and decreased MHC-I molecules in PSMB10-OE THP-1 cells compared with those in shCTRL THP-1 cells (Fig. 6A and B). Next, we found that *PSMB10* KD enhanced the T-cell killing of THP-1 cells, while the overexpression of *PSMB10* effectively rescued this process (Fig. 6C and D). Further co-IP assay in THP-1 cells revealed a direct interaction between PSMB10 and MHC-I molecules (Fig. 6E). To determine the mechanism by which PSMB10 downregulates MHC-I molecules, shPSMB10-transfected THP-1 cells were treated with CHX for 0, 4, or 8 h. The levels of MHC-I were then analyzed using WB analysis (Fig. 6F). We found that the downregulation of PSMB10 led to increased MHC-I levels, not through protein synthesis regulation. However, ubiquitination assays demonstrated decreased ubiquitination and elevated levels of MHC-I protein in shPSMB10-transduced THP-1 cells compared to shCTRL-transduced THP-1 cells (Fig. 6G). These results suggest that PSMB10 degrades MHC-I proteins via ubiquitination in AML cells.

To further confirm the conclusion, we used a shβ2-microglobulin (β2m) lentivirus to abrogate β2m, one of two polypeptide chains of MHC-I molecules [42, 43], in shPSMB10-transfected THP-1 cells and cocultured them with activated healthy human T cells. The results showed that β2m KD led to decreased MHC-I molecule levels (Fig. 6H and I) with decreased CTL killing of leukemia cells (Fig. 6J), suggesting that increased PSMB10 results in leukemia cell resistance to CTL-mediated killing through the direct interaction and ubiquitinated degradation of MHC-I protein.

### *Psmb10 *is dispensable for normal hematopoiesis

**Fig. 7:**
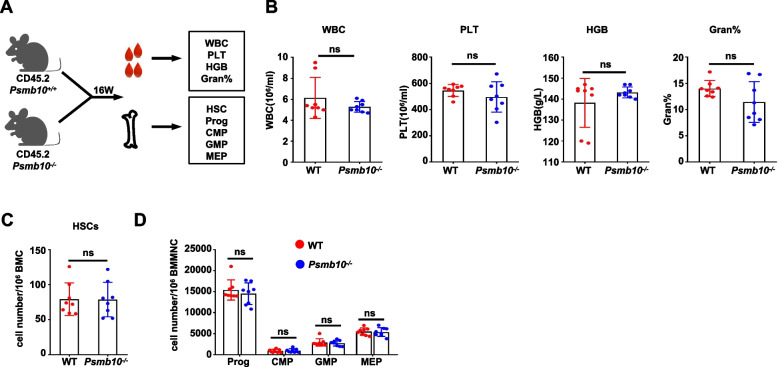
*Psmb10* is dispensable for normal hematopoiesis. **A** Experimental scheme for B-D. Created with figdraw.com. **B** Numbers of WBC, platelet, HGB, and neutrophilic granulocyte percentage (Gran%) in PB of 16-week-old *Psmb10*^*−/−*^ or *Psmb10*^+/+^ control C57BL/6 J mice (*n* = 8). **C-D** Immunophenotypic quantification of HSC abundance (HSCs: Lin^−^Sca1^+^cKit^+^CD48^−^CD150^+^) (**C**), progenitor cells (Prog: Lin^−^Sca1^−^ c-Kit^+^), CMPs (Lin^−^Sca1^−^c-Kit^+^CD34^+^ CD16/32^−^), GMPs (Lin^−^Sca1^−^ c-Kit^+^ CD34^+^ CD16/32^+^) and MEPs (Lin^−^Sca1^−^c-Kit^+^CD34^−^CD16/32^−^) (**D**) in the BM of 16-week-old *Psmb10*^*−/−*^ or *Psmb10*^+*/*+^ control C57BL/6 J mice (*n* = 8). BMC: bone marrow cell; BMMNC: bone marrow mononuclear cell; CMP: common myeloid progenitors; HGB: Hemoglobin; HSC: hematopoietic stem cell; GMP: granulocyte–macrophage progenitors; gran: granulocytes; MEP: megakaryocyte-erythroid progenitors; PLT: platelet; WBC: white blood count; WT: wild-type. ns, not significant. The error bars denote the means ± SDs

To identify whether *Psmb10* could act as a special molecular target for the eradication of drug-resistant LSCs, we examined its effects on normal hematopoiesis in 16-week-old *Psmb10*^−/−^ mice with steady-state hematopoiesis (Fig. 7A). Compared with *Psmb10*^+/+^ mice, *Psmb10*^−/−^ mice presented similar blood indices, including white blood cell counts, platelet counts, hemoglobin levels, and the percentage of granulocytes (Fig. 7B). Furthermore, the percentages of HSCs, hemopoietic progenitor cells, common myeloid progenitors, granulocyte–macrophage progenitors, and megakaryocyte-erythroid progenitors in the BM of *Psmb10*^−/−^ mice were similar to those in the BM of *Psmb10*^+/+^ mice (Fig. 7C and D), indicating that *PSMB10* is dispensable for normal hematopoiesis.

## Discussion

Previous studies have suggested that restarting cellular senescence [6] combined with immune killing [19, 22] could be an effective way to eradicate LSCs. Here, we found that there was a 13-fold increase in PSMB10 mRNA expression in surviving nonsenescent LSCs compared to senescent LSCs following chemotherapy. The genetic inactivation of PSMB10 results in increased senescence initiation, CTL killing, and chemotherapeutic drug-induced senescence in different types of human AML cells. Furthermore, the downregulation of PSMB10 hindered the initiation of leukemia and the maintenance of LSC with a 19-fold reduction in the frequency of human LSCs in the human-to-mouse leukemia cell transplantation model. It also promoted the chemotherapeutic drug-mediated eradication of LSCs with a 7.6-fold decrease in drug-resistant LSCs in the syngeneic mouse leukemia cell transplantation model, while leaving normal hematopoietic cells unaffected. Thus, we here identified the especially increased PSMB10 in LSCs as a new key stemness maintenance molecule for chemotherapeutic drug-resistant LSCs.

Despite the increasing understanding of the molecular mechanisms underlying chemoresistance, only a limited number of clinical prognostic biomarkers have been developed to predict patient outcomes and guide therapeutic decision-making [44]. Previous studies have identified some genetic abnormalities, epigenetic profiles, and protein expression patterns that may serve as valuable diagnostic and prognostic biomarkers for AML [45], which include mixed lineage leukemia (MLL) fuse genes and FLT3-ITD mutations, epigenetic factors such as DNMT3A and IDH, proteins molecules such as UBA1 and FIBA, etc. [46–49]. Here, we found that the markedly elevated expression of PSMB10 in LSCs is closely associated with poor therapeutic response and prognosis in patients with AML. Thus, we propose that the especially increased PSMB10 in LSCs could serve as a new novel biomarker for predicting therapeutic reaction and prognosis in AML patients.

Previous studies found that the stemness of LSC is decided via differentiation block, resistance to apoptosis and senescence, as well as immune escape, etc. [19, 50–53]. The important thing is that LSC in APL could be eradicated via arsenic trioxide-induced promyelocytic leukemia-p53 axis activation-mediated senescence [6–8]. However, the leukemic cells have marked heterogeneity, and it remains undiscovered for the mechanism by which the LSCs from chemotherapeutic drug-resistant patients with AML, especially non-APL, maintain stemness via senescent and immune regulation. Here, we found that the especially increased PSMB10 in non-senescent drug-resistant LSC acts as a key molecule not only for resistance to senescence induction and initiation but also for escape from CTL killing, leading to their stemness maintenance in patients with relapsed and refractory AML. Although a recent study revealed that the immunoproteasome subunit PSMB8 maintains oncogenic gene expression in KMT2A complex-driven leukemia [54], we first found that the increased PSMB10 but not PSMB8 is involved in chemotherapeutic drug-resistant LSC maintenance via senescent and immune regulation.

Our further molecular mechanism studies found that PSMB10 downregulated both RPL6 and RPS6 in a ubiquitination-mediated degradation manner. Previous studies revealed that RPS6 phosphorylation promoted the initiation of protein synthesis following mitotic or nutritional stimulation [55] and that RPL6 inhibited the ubiquitin ligase activity of MDM2 by directly binding to MDM2 [39]. Here, we uncovered previously unknown mechanisms by which the downregulation of RPs expression in leukemia cells enhances the translation of MDM2 via increased RPL6-mediated translation efficacy and RPS6-mediated translation initiation, and simultaneously leads to a decreased direct bind to MDM2 protein in the complex form of both RPL6 and RPS6 proteins, which further promotes the MDM2-mediated ubiquitin-independent degradation of P21 protein. It was also found that the increased PSMB10 inhibited the chemotherapy drug-mediated senescence induction by impeding drug influx via the ubiquitin-mediated degradation of SLC22A16. This mechanism differs from previous findings, which identified drug resistance in AML as primarily resulting from the increased activity of ATP-dependent efflux pumps that expel various drugs from AML cells [56, 57]. Since chemotherapy induces the senescence of leukemia cells partly via P21 [58], combined with our discovery that PSMB10 induces MDM2-mediated degradation of P21 protein, we conclude that the increased PSMB10 also impedes chemotherapy drug-induced P21-dependent senescence of AML cells.

The ability to evade immune killing is another critical mechanism by which AML cells preserve their stemness [19, 22]. The immunoproteasomes have been shown to generate peptides presented by MHC-I molecules, which were then recognized by CD8^+^ T cells for tumor immune surveillance [13, 59]. Research indicated that PML/RARα suppresses activation of the immunosubunits in APL, which may be critical for malignant cells to escape immune recognition [60]. Previous studies also suggested that the absence or dysfunction of immunoproteasomes results in immune escape in some solid tumor cells due to the inability of antigen processing and presentation [61–63]. In contrast to these acknowledged functions to facilitate the immune clearance of tumor cells, we revealed here that the increased PSMB10 instead promotes AML cells to escape from CTL-killing both in vitro and in vivo through ubiquitinated degradation of MHC-I proteins.

Although targeted therapies, such as BCL-2, FLT3, and IDH inhibitors, as well as CD33 antibody–drug conjugates and oral hypomethylating agents, have shown significant benefits in the treatment of AML [64], targeted therapy for AML remains challenging because LSC could not get effective targeted clearance by currently available treatments. The PSMB10 targeted therapy for LSC may provide new hope because the especially increased PSMB10 in LSC has the following advantages: first, the immunoproteasome is especially expressed in the hematopoietic and lymphoid tissues [65], and we confirmed that it is dispensable for normal hematopoiesis (Fig. 7). This suggests that PSMB10, with its specificity for LSCs, is a safe target for LSC-targeted therapy. Moreover, it has been confirmed that the significantly elevated levels of PSMB10 act as a key molecule in maintaining stemness in drug-resistant AML LSCs through mechanisms related to senescence and immune regulation.

Although our results suggested that in the mouse model, PSMB10 downregulation sensitized AML cells to AraC/ADM treatment and enhanced the in vivo clearance of drug-resistant LSCs by increased intracellular drug concentrations and drug-induced cellular senescence, as well as enhanced cytotoxic T lymphocyte-mediated killing in a ubiquitinated degradation manner, while normal hematopoietic cells remained unaffected. However, non-selective proteasome inhibitors, such as first-generation boronic acid bortezomib [66] and the second-generation epoxyketone carfilzomib [67, 68], are lack of selectivity for constitutive proteasome and immunoproteasome, which target more than one composition subunit at a time and therefore are considered broad-spectrum proteasome inhibitors. Their clinical utility will lead to severe off-target toxicity such as neuropathy, hematologic toxicity and cardiac effects, etc. [69]. LU-002i, a β2i-selective inhibitor, exhibits a defined bioactive configuration [70]. But β2i-selective inhibitor remains unexplored in preclinical studies, highlighting an area for future investigation. Thus, the selective PSMB10 inhibitor combined with chemotherapy could be a new effective and safe treatment mode for eradication of drug-resistant AML LSCs.

Other studies have shown that BM microenvironment cells, such as mesenchymal stem cells and adipocytes, were also involved in stemness maintenance and drug resistance in AML cells [9, 10, 71–74]. Therefore, whether there is crosstalk between PSMB10 and the BM microenvironment is worthy of further investigation. Moreover, it is still unclear whether there are other key signaling molecules or pathways involved in the maintenance of the stemness of drug-resistant LSCs via the regulation of senescence and immune escape. Besides, our results showed that PSMB10 degraded RPL6/RPS6, SLC22A16, and MHC-I protein molecules in a ubiquitin–proteasome manner, but whether and how these degradations were selective remain to be investigated in the future.

## Conclusion

**Fig. 8:**
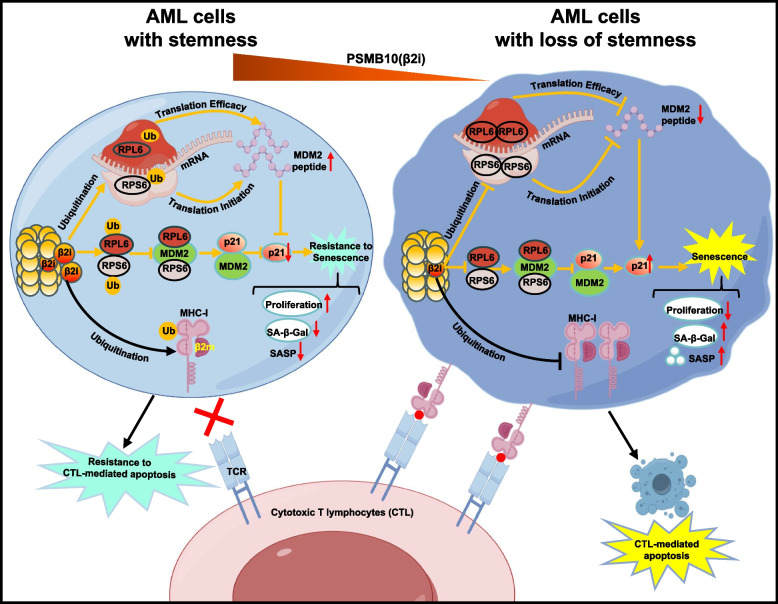
A proposed mechanism for the increased PSMB10 to maintain the stemness of drug-resistant leukemia cells. Created with figdraw.com. PSMB10 is significantly upregulated in chemotherapeutic drug-resistant LSCs, leading to the downregulation of both RPL6 and RPS6 proteins through ubiquitination-mediated degradation. Then the decreased RPL6 and RPS6 proteins, on the one hand, result in an increased MDM2 protein via the upregulation of translation activity, on the other hand, lead to a decreased RPs complex binding-induced conformational change of MDM2, which further promotes the MDM2-mediated ubiquitin-independent degradation of P21^Waf1^ protein and resistance to senescence in AML cells. Besides, the increased PSMB10 also induces leukemia cell resistance to CTL-mediated killing by a direct binding and the ubiquitinated degradation of MHC-I proteins. AML: acute myeloid leukemia; Ub: ubiquitination; SASP: senescence-associated secretory phenotype; CTL: cytotoxic T lymphocyte; TCR: T-cell Receptor; β2m: β2-microglobulin; MHC-I: major histocompatibility complex class I

To sum up, this study identified the markedly increased PSMB10 as a promising candidate target molecule for addressing chemotherapy-resistant LSCs via senescent induction and further immune eradication (Fig. 8). Targeting PSMB10, in combination with chemotherapy and adoptive T-cell therapy, could represent a novel approach to promote the cure of patients with refractory and relapsed AML.

## Supplementary Information

Supplementary Material 1.

## Data Availability

The mass spectrometry proteomics data have been deposited to the ProteomeXchange Consortium via the iProX partner repository [75, 76] with the dataset identifier PXD058714.

## Abbreviations

ADM: Adriamycin
AML: Acute myeloid leukemia
APL: Acute promyelocytic leukemia
AraC: Arabinoside Cytosine
BM: Bone marrow
BMMNC: Bone Marrow Mononuclear Cell
BMT: Bone marrow transplantation
β2m: β2-Microglobulin
CHX: Cycloheximide
CMP: Common myeloid progenitors
CTL: Cytotoxic T lymphocyte
CR: Complete Remission
co-IP: Coimmunoprecipitation
DNR: Daunorubicin
E: T: Effector/target ratios
FCM: Flow cytometry
GMP: Granulocyte–macrophage progenitors
GFP: Green Fluorescent Protein
Gran: Granulocyte
HGB: Hemoglobin
HPC: Hemopoietic progenitor cell
HSC: Hematopoietic stem cell
hCD45^+^: Human CD45^+^
KD: Knock down
KO: Knock out
LSC: Leukemic stem cell
MA9: MLL-AF9
MEP: Megakaryocyte-erythroid progenitors
MFI: Mean fluorescence intensity
MHC-I: Major Histocompatibility Complex Class I
NR: No Response
NCG: NOD/ShiLtJGpt-*Prkdc*^em26Cd52^*Il2rg*^em26Cd22^/Gpt
OE: Overexpression
PPI: Protein‒protein interaction
PB: Peripheral blood
PLT: Platelet
Prog: Progenitor cell abundance
RP: Ribosomal protein
RPL6: Ribosomal protein L6
RPS6: Ribosomal protein S6
SASP: Senescence-associated secretory phenotype
SD: Standard Deviation
SiNC: Small interfering RNA negative control
shRNA: Short hairpin RNA
shCTRL: Non-targeting control
SA-β-gal: Senescence-associated β-galactosidase
TCR: T-cell Receptor
TCGA: The Cancer Genome Atlas
Ub: Ubiquitination
WBC: White blood count
WB: Western blot
WT: Wild-type
