# Supplementary material for: PSMB10 maintains the stemness of chemotherapeutic drug-resistant leukemia cells by inhibiting senescence and cytotoxic T lymphocyte-mediated killing in a ubiquitinated degradation manner
Source: J Exp Clin Cancer Res. 2025 Jun 3;44:170. doi: 10.1186/s13046-025-03420-9 (PMC12131556; doi:10.1186/s13046-025-03420-9)
Supplement: Supplementary file 1 — Supplementary Material 1. [file 13046_2025_3420_MOESM1_ESM.doc]

**Supplementary Table 1: Sequences of shRNAs and siRNAs**

| Name | Sequences |
| --- | --- |
| PSMB10 shRNA | ATACGCGAGCCACTAACGATT |
| Control shRNA | TTCTCCGAACGTGTCACGT |
| PSMB10 siRNA | AUACGCGAGCCACUAACGAUUTT |
| NC siRNA | UUCUCCGAACGUGUCACGUTT |
| RPL6 shRNA | CAGAAAGCTGTGGACTCACA |
| Control shRNA | TTCTCCGAACGTGTCACGT |
| RPS6 shRNA | GGAGAAGCGCCAGGAACAAAT |
| Control shRNA | TTCTCCGAACGTGTCACGT |
| β2m shRNA | GAATGGAGAGAGAATTGAA |
| Control shRNA | TTCTCCGAACGTGTCACGT |

**Supplementary Table 2: The primers used for the quantitative qPCR**

| Symbol gene | Primer sequences (Forward) | Primer sequences (Reverse) |
| --- | --- | --- |
| h-GAPDH | ACCCACTCCTCCACCTTTGAC | TCCACCACCCTGTTGCTGTAG |
| h-PSMB10 | TGGCGTCCAAGATGGAGCTA | GGGGATGCACACCGTAGAG |
| h-P21 | CACTCCAAACGCCGGCTGATCTTC | TGTAGAGCGGGCCTTTGAGGCCCTC |
| h-MMP9 | GATCCAGGTGGGTAGAAGGTC | CCCCTGCAAACTTCGTCCT |
| h-IL6 | GGTACATCCTCGACGGCATCT | GTGCCTCTTTGCTGCTTTCAC |
| h-IL8 | TTTTGCCAAGGAGTGCTAAAGA | AACCCTCTGCACCCAGTTTTC |
| h-PAI1 | GGCTGGTGCTGGTGAATGC | TCGGGCGTGGTGAACTCAG |
| NF-kB | ATGTGGAGATCATTGAGCAGC | CCTGGTCCTGTGTAGCCATT |
| h-CDK2 | CGGACAAATAAAACTCGCTGACTTC | TGGACTGGAGCAAGACTTCGG |
| h-CDK4 | TGAAATTGGTGTCGGTGCCTATG | TCCAGTCGCCTCAGTAAAGCC |
| h-CDK6 | CGGACAAATAAAACTCGCTGACTTC | TGGACTGGAGCAAGACTTCGG |
| h-Cyclin D1 | GCTGCGAAGTGGAAACCATC | CCTCCTTCTGCACACATTTGAA |
| h-Cyclin E2 | TCAAGACGAAGTAGCCGTTTAC | TGACATCCTGGGTAGTTTTCCTC |
| h-MDM2 | GGCAGGGGAGAGTGATACAGA | GAAGCCAATTCTCACGAAGGG |

**Supplementary Table 3: The marker genes for HSCs and LSCs identification**

| HSCs | LSCs |
| --- | --- |
| SPINK2 ZFAS1 NRIP1 GAS5 JUN MEIS1 HLF EGR1 CRHBP NPR3 FAM30A MMRN1 MSI2 ANGPT1 EIF4A2 TPT1 COMMD6 EEF1A1 SNORD13 FAU FOS PHLDB2 MSRB3 GUCY1A3 C6orf48 GBP4 PCDH9 SNHG6 RACK1 ADGRG6 PREX2 PRKG2 HINT1 RNA5-8S NFKBIZ MYCT1 TUBA1A CD34 ZBTB20 TFPI SNHG8 AVP CEP70 H3F3B RNU4-2 HIST1H2BG HOPX TSC22D1 LRRC70 PBX1 | SPINK2 ANGPT1 GUCY1A3 FAM30A MMRN1 TPT1 GAS5 RAB27B TPM4 MSI2 GCSAML SOCS2 EEF1A1 NRIP1 HOPX CD34 TFPI TPSD1 PDZRN4 PCNP PTPRCAP FLT3 SMIM24 SELENOP DAPK1 SMYD3 ADGRG6 PIM1 MECOM CEP70 XIRP2 SPAG6 TAPT1-AS1 GNA15 DSE TPSAB1 TPSB2 H2AFY SCHIP1 LINC02470 NPR3 KMT2A CD200 MACF1 GBP4 ABCC1 PROM1 TMEM70 FAM110A TMEM123 |

**Supplementary Figures**

**Supplementary Figure. S1 The specific increased expression of PSMB10 in post-chemotherapy nonsenescent LSCs predicts a poor AML prognosis. (A)** *PSMB10* mRNA levels in the HSCs and LSCs of AML patients from the single-cell RNA-seq dataset EGAD00001008373. **(B)** Relative *PSMB10* mRNA levels between AML HSCs and LSCs and between two different LSC groups at diagnosis and complete remission (CR) from the same AML patient, which were analyzed with data (GSE185991) from single-cell RNA-seq. **(C)** Relative *PSMB10* mRNA levels in the BM mononuclear cells of patients with different FAB subtypes from TCGA samples. LSCs: leukemia stem cells; HSCs: hematopoietic stem cells. FAB: French-American-British. ***p* < 0.01, *****p* < 0.0001 (t test). The error bars denote the means ± SD.

**Supplementary Figure. S2 Downregulation of PSMB10 restarts senescence of AML cells without apoptosis *in vitro*.** **(A)** Immunoblot showing the KD efficacy of PSMB10 in THP-1 and KG-1a cells. **(B)** Representative FCM images of the cell cycle distribution in THP-1 and KG-1a cells after transduction with the indicated lentiviruses. **(C)** Representative FCM images and statistical histogram of EdU^+^ percentage of THP-1 and KG-1a cells after transduction with the indicated lentiviruses. **(D)** Distribution of Annexin V^+^ apoptotic cells in THP-1 and KG-1a cells after transduction with the indicated lentiviruses. PI: Propidium Iodide. ***p* < 0.01, and ****p* < 0.001 (t test). ns, not significant. The error bars denote the means ± SDs.

**Supplementary Figure. S3 Loss of PSMB10 boosts chemotherapy-induced senescence *in vitro* and eradication of drug-resistant LSCs *in vivo*. (A)** Relative mRNA levels of SASP- and cell cycle-related genes in THP-1 and KG-1a cells transduced with the indicated lentiviruses under AraC treatment. **(B)** Changes in the protein levels of CDK2, CDK4, CDK6, CCNE2, Phospho-Rb and P21 in THP-1 and KG-1a cells transduced with the indicated lentiviruses under AraC treatment. **(C)** Percentages of GFP^+^ leukemia cells in PB at the indicated times after the 1^st^ BMT before chemotherapy (n=6). **(D)** Percentages of GFP^+^ leukemia cells in the PB after the death of 1^st^ BMT recipient C57BL/6J mice on day 21 (n=6). **(E)** Spleen size and percentage of GFP^+^ leukemia cells in the spleen after the death of 1^st^ BMT recipient C57BL/6J mice on day 21 (n=6). **(F)** Percentage of GFP^+^ leukemia cells in the PB after the death of 2^nd^ BMT recipient C57BL/6J mice at 4 weeks (n=6). **(G)** Statistical histograms of the percentage of CD93^+^ LSCs after the death of 2^nd^ BMT recipient C57BL/6J mice at 4 weeks (n=5-6). AraC: Arabinoside Cytosine; ADM: Adriamycin; PB: peripheral blood; GFP: green fluorescent protein. **p* < 0.05, ***p* < 0.01, ****p* < 0.001 and *****p* < 0.0001 (t test). ns, not significant. The error bars denote the means±SDs.

**Supplementary Figure. S4 PSMB10 impedes RPL6/RPS6-MDM2-P21 senescence pathway and SLC22A16-mediated drug endocytosis in AML cells. (A)** Changes in the protein levels of P21, CDK4, CCND1, Lamin B1 and PSMB10 in si-PSMB10- and si-NC-transfected THP-1 cells. **(B)** Relative mRNA levels of *P21*, *CDK4* and *CCND1* genes in si-PSMB10- and si-NC-transfected THP-1 cells. **(C)** A PPI network was generated by mapping significantly differentially expressed proteins related to the senescence proteome in PSMB10-KD THP-1 cells. (**D**) Quantification analysis of ubiquitinated RPL6 and RPS6 normalized to GAPDH**. (E)** RT‒qPCR analysis of MDM2 mRNA in shCTRL- and shRPL6-transduced THP-1 cells with shPSMB10. **(F)** RT‒qPCR analysis of MDM2 mRNA in shCTRL- and shRPS6-transduced THP-1 cells with shPSMB10. **(G)** Scheme showing the method for assessing MDM2 translation initiation efficacy. **(H)** DNR influx and efflux kinetics in shCTRL- and shPSMB10-transduced THP-1 cells. **(I)** Representative FCM images **(left)** and statistical histogram **(right)** of intracellular daunorubicin in shCTRL- and shPSMB10-transduced THP-1 cells cultured in the presence of 0.25 µg/ml daunorubicin for 1 h. **(J)** Statistical histogram of the cumulative rate of decrease in intracellular daunorubicin fluorescence in shCTRL- and shPSMB10-transduced THP-1 cells at the indicated times. **(K)** SLC22A16 protein levels in shCTRL- and shPSMB10-transduced THP-1 cells. **(L)** Immunoprecipitation assay of PSMB10 and SLC22A16 in THP-1 cells. **(M)** shPSMB10 cells were treated with CHX at the indicated times, and SLC22A16 protein levels were detected via WB analysis. **(N)** Ubiquitination assays of SLC22A16 in lysates from shCTRL- or shPSMB10-transduced THP-1 cells. MFI: Mean fluorescence intensity; DNR: Daunorubicin; WT: wild-type; CHX: cycloheximide; Ub: ubiquitination. ****p* < 0.001, *****p* < 0.0001 (t test). ns, not significant. The error bars denote the means ± SDs.
